# Integrating multi-source remote sensing, field work, and petrography for automatic lithological mapping and mineralization potential in the Gabal El-Faraid, Egypt

**DOI:** 10.1038/s41598-026-55916-9

**Published:** 2026-06-04

**Authors:** Hassan Osman, Mokhles K. Azer, Khairy S. Zaky, Shehata Ali, Esam S. Farahat, Saif M. Abo Khashaba

**Affiliations:** 1 Geology Department, Faculty of Science, Minia University, El-Minia, 61519 Egypt; 2 Geological Sciences Department, National Research Center, Cairo, Egypt; 3 Geology Department, Faculty of Science, Kafrelsheikh University, Kafrelsheikh, 33516 Egypt

**Keywords:** Hyperspectral PRISMA, Machine learning, Fuzzy-Logic, ANS, Mineral potential, Petrography, Shear zones, Environmental sciences, Solid Earth sciences

## Abstract

The Gabal El-Faraid Group, in the Egyptian Southeastern Desert, comprises Neoproterozoic lithologies and structurally controlled mineralization zones that require an integrated exploration approach. This study integrates multi-source remote sensing data with detailed field observations, structural analysis, and petrographic investigations to improve lithological differentiation and delineate mineralization-related structures. Results indicate a potential structural-control spatial association among shear zones, hydrothermal alteration zones, and mineralization, suggesting their potential role in controlling mineralization. Support Vector Machine (SVM) and Random Forest (RF) algorithms have been used to best delineate lithological rock units of the study area. Accurate lithological mapping was achieved using an SVM applied to combined Sentinel-2 and ALOS PALSAR datasets, yielding an overall accuracy of 92.67%, a Kappa coefficient of 0.8928, and an F1-score of 88.64%. Six spectral mineral indices, like argillic, clay, ferrous silicates, ferrugination, hydroxyl, and phyllic alterations, have been emphasized using the hyperspectral PRISMA dataset. Exposed rocks comprise metavolcanics, metagabbro-diorite, tonalite-granodiorite, monzogranite, syenogranite, pegmatite, and late basic dikes, representing a multiphase magmatic sequence spanning arc-related to post-collisional stages. Structural investigations indicate a polyphase deformation history comprising four major events (D1-D4). The earliest phase (D1) is characterized by penetrative foliation, stretching lineation, and low-angle thrusting associated with nappe transport during intra-oceanic arc accretion. D2 reflects large-scale folding and regional shortening related to arc-to-continent collision. The D3 phase records transpressional strike-slip shearing, reactivating earlier structures and facilitating magma ascent, whereas D4 is marked by brittle faulting and jointing linked to post-orogenic uplift and extensional tectonics. Lineament analysis from PALSAR data highlights dominant NW-SE and NE-SW trends that closely correspond to field-measured fabrics and shear zones. Integration of field and remote sensing datasets demonstrates that late-stage deformation (D3-D4) exerted strong structural control over pegmatite emplacement, quartz veining, and hydrothermal alteration, with a main set trending WNW to NW and a less prominent set with pronounced ENE and NNW trends, emphasizing the role of inherited Pan-African structures in localizing mineralization. High-priority prospective zones in the studied granites were derived from integrated datasets, which were systematically combined in ArcGIS using a fuzzy-logic overlay. The detected zones are labeled as very high, high, moderate, low, and very low based on their potential for rare-metal mineralization. The integrated datasets can be applied to similar areas in the Eastern Desert of Egypt and across the entire Arabian-Nubian Shield (ANS).

## Introduction

Granites and associated pegmatites represent some of the most significant global repositories of strategic minerals and rare earth elements (REEs), commonly exhibiting enrichment in a variety of other critical metals^1–5^. The rapid expansion of industrial, technological, and energy-related applications for these elements has intensified scientific interest in pegmatite systems and their associated mineralization. Over recent decades, increasing attention has been given to the geological characteristics, compositional variations, mineral assemblages, and isotopic signatures of granitic pegmatites, which collectively indicate strong genetic links to their parental granites^6,7^. In Egypt, mineralization is predominantly associated with younger granites and their pegmatitic offshoots^8,9^. These granites, ranging from post-orogenic to anorogenic types, constitute an important exploration target for REEs and related strategic elements. Numerous granitic pegmatite occurrences have been documented across the Eastern Desert, including Wadi Zareib, Rod Ashab, Gabal Um Anab, Gabal Ras Baroud, Gabal El Sibai, El-Nikeiba, Umm Naggat, and Gabal Abu Dob^10–15^. Additional distinctive pegmatite bodies are reported in the southeastern Desert (SED)^15–18^.

Remote sensing techniques are crucial for mineral exploration, providing accurate, cost-effective lithological and mineral mapping, especially in arid and semi-arid regions, as an alternative to traditional rock identification, which relies on time-consuming, expensive fieldwork and lab-based methods and is constrained by limited spatial extent^5,19,20,21^. Optical remote sensing data, such as Sentinel-2, enable distinguishing lithological units by detecting subtle spectral differences across geological formations, which is essential for accurate mapping across varied terrains^5,19,22–28^. However, to resolve complex terrains, recent studies have increasingly leveraged sensor fusion. Integrating optical datasets with Synthetic Aperture Radar (SAR), such as ALOS PALSAR, enhances classification strength, particularly when coupled with machine learning algorithms such as Support Vector Machines (SVM) and Random Forest (RF)^29–31^.

Mapping altered zones in mineralized granites, which typically exhibit characteristic patterns, can enable rapid, cost-effective identification of promising areas^5,19,24,32–35^. Despite advances in multispectral datasets, broadband sensors often lack the spectral resolution required to detect alteration zones, which are essential for mineral prospectivity mapping. The advent of spaceborne hyperspectral imagery, specifically PRISMA, addresses this gap by capturing continuous spectral data across the 400–2500 nm range. This high-fidelity spectral profiling facilitates the detection of diagnostic absorption features, enabling more accurate differentiation of alteration zones compared to multispectral datasets^20,36^.

Distinguishing high-potential zones within the prospected area is a key objective in mineral exploration. Over the last few decades, mineral exploration research has developed a wide range of knowledge- and data-driven techniques for integrating multiple evidential layers to generate reliable prospectivity maps for targeted mineralization types^37,38^. In this study, fuzzy overlay analysis is a GIS-based multi-criteria integration technique that combines multiple evidential layers while explicitly accounting for uncertainty and gradual transitions between favorable and unfavorable conditions. Fuzzy logic assigns continuous membership values from 0 to 1, thereby better reflecting the inherent ambiguity of geological, geochemical, and remote-sensing data. Fuzzy operators such as fuzzy AND, OR, PRODUCT, SUM, and GAMMA can be used to combine criteria based on their importance and their interactions. Fuzzy overlay is especially well-suited for mapping mineral prospectivity, as it can handle features such as lithology, alteration, and structure that often lack clear boundaries. As a result, fuzzy overlay has been widely used in mineral exploration investigations to make predictions more accurate and decisions less subjective^39–41^.

The geology, structural framework, mineralogy, and geochemistry of the Gabal El-Faraid area have been studied by many researchers (e.g., 8, 19–25). These studies have been focused mainly on mineralization potential, as well as on the geochemical and radiometric features of the granites and pegmatites associated with them. However, comparatively little attention has been given to the detailed structural framework that controls the emplacement, distribution, and deformation of these rocks. Therefore, the spatial relationships between structural elements and granitic intrusions remain poorly constrained. Moreover, a comprehensive and up-to-date geological map of the area integrating recent field observations with modern analytical approaches is still lacking. Therefore, there is a lack of understanding of the structural setting controlling the distribution of REE-bearing pegmatites and critical metal mineralization in granites.

This study provides integration of multisource remote sensing datasets, detailed field observations, petrographic analyses, and structural mapping. The main contributions of these integration approaches in the present study include: (1) accurately lithological mapping using machine learning SVM and RF classifiers, (2) delineate mineralized alteration zones using hyperspectral PRISMA as well as detect surface structure lineaments using ALOS PALSAR radar dataset, (3) mineral potential mapping using fuzzy logic overlay within the G. El-Faraid area, and reconstruction of the deformation history and tectonic evolution of the area.

## Geologic setting

**Fig. 1:**
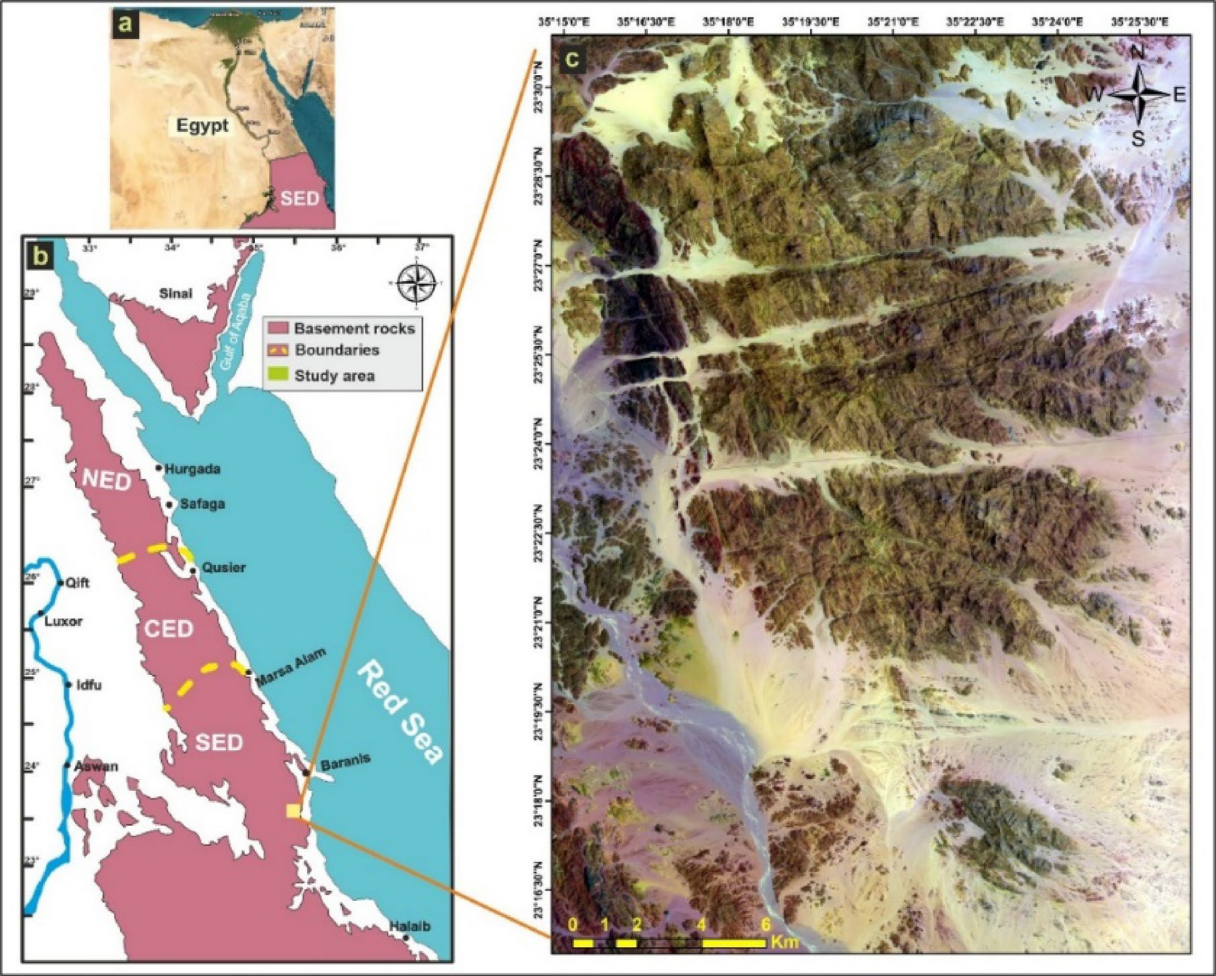
**a**) shows the location of Egypt, **b**) shows the eastern desert map and highlights the distribution of Precambrian basement rocks, **c**) shows the location of G. El-Faraid Group NE-Wadi El-Rahaba area, using a Landsat-8 OLI false color composite (7-5-3) in RGB showing the main lithological units of the study area. The figure was created by ArcGIS Desktop 10.8. (https://www.esri.com/enus/arcgis/products/arcgis-desktop/overview/).

The study area lies within the Eastern Desert of Egypt, the northern extension of the Arabian Nubian Shield (ANS) (Fig. 1a, b), a vast Neoproterozoic assemblage of juvenile crust that formed during the East African Orogeny^14,42^. The tectonic evolution of the ANS involved a transition from oceanic subduction and arc accretion to continental collision between East and West Gondwana at around 630 Ma, followed by post-orogenic collapse and extensional rifting during the Ediacaran to early Cambrian^43,44^. In the Eastern Desert, this evolution is recorded by an older assemblage of ophiolites, metavolcanic sequences, and gabbro^45,46^. Within the study area, the G. El-Faraid granite intrudes older arc-related complexes and metasedimentary rocks and is internally undeformed, consistent with post-tectonic emplacement following the main Pan-African shortening during Ediacaran collapse.

The G. El-Faraid group is located between latitudes 23°30’- 23°37’ N and longitudes 35°15’-35°25’ E. It is located about 28 km north of El-Shalateen city in the SED (Fig. 1c). The area exhibits generally low to moderate relief, with granitic hills forming prominent peaks reaching up to 1366 m, while the lowest elevations are about 120 m above sea level.

**Fig. 2:**
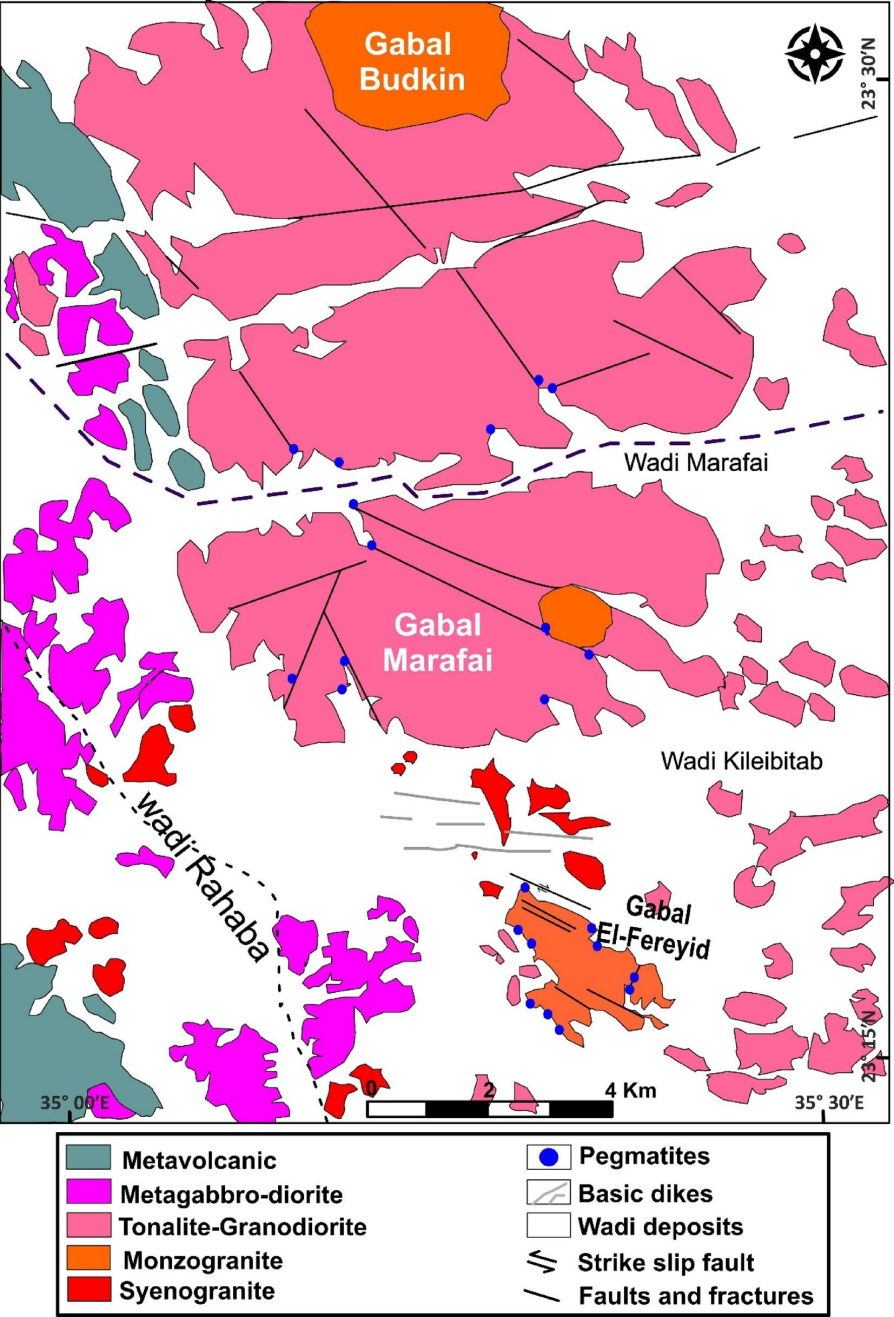
Geological map of G. El-Faraid, group after^47^. The figure was created by ArcGIS Desktop 10.8. (https://www.esri.com/enus/arcgis/products/arcgis-desktop/overview/).

The Precambrian basement rocks exposed in this area along northeast Wadi El-Rahaba represent a part of the Pan-African belt of the ANS, comprising the following from oldest to youngest: Precambrian infrastructural rock associations comprising gneisses that crop out in the northeastern and southwestern parts of the area, ophiolite, and tonalite, granodiorite, both of which are intruded by monzogranite and syenogranite, and finally, all these rock units are intruded by basic dikes and pegmatite, respectively (Fig. 2)^47^.

**Fig. 3:**
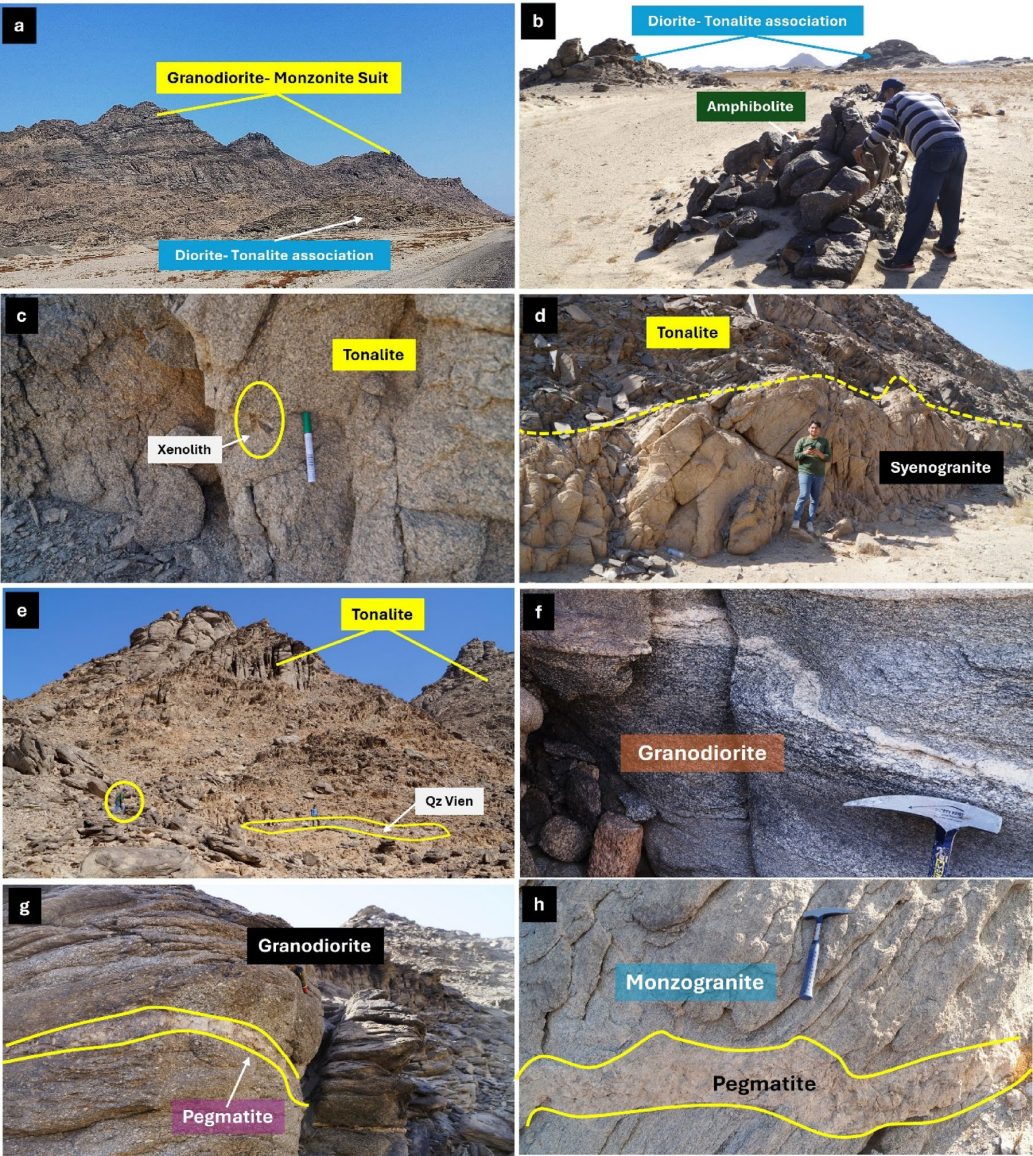
Field photographs at G. El-Faraid group showing **a**) highly foliated diorite-tonalite intruded by Granodiorite-Monzonite suite, **b**) highly deformed amphibolite, **c**) Close-up view showing tonalite containing xenoliths, **d**) Tonalite intruded by Synogranite makes sharp contact with granite, (**e**) highly deformed milky quartz vein with tonalite, (**f**) foliated granodiorite contain pegmatite vein, and **h**) close-up view of monzogranite contain pegmatite vein. All photographs were taken by the authors during field investigations. Written informed consent for publication was obtained from all identifiable individuals.

Tonalite-Granodiorite assemblage yields crystallization ages of ~ 760 − 720 Ma based on U-Pb zircon geochronology (SHRIMP and LA-ICP-MS), corresponding to early Pan-African arc-related magmatism in the ANS^45^. crops out mainly in the central part of the area as whitish-green to grey, medium- to coarse-grained hornblende diorites and biotite tonalites. This assemblage forms moderate-relief hills and highly exfoliated, deeply weathered low, detached hills, and locally contains abundant xenoliths of metagabbro and amphibolite. Diorite-tonalite association intrudes the older metagabbro-diorite complex, with sharp, well-defined intrusive contacts (Fig. 3a, b).

Tonalite represents the principal lithological unit of the study area, is typically medium- to coarse-grained, and exhibits grey to greyish-green colors. It is commonly highly exfoliated and deeply weathered detached hills that locally encircle the margins of younger monzogranite plutons. The tonalite commonly encloses subrounded xenoliths of diorite and amphibolite reaching up to ~ 30 cm in diameter (Fig. 3c). The tonalite are subsequently intruded by younger monzogranite and synogranite plutons, which display clear intrusive contacts with the host tonalite (Fig. 3d). Highly deformed milky quartz vein widely found with tonalite(Fig. 3e). These field relationships reflect a multiphase magmatic evolution, in which tonalite represents the earliest major intrusive phase, followed by felsic granitoid magmatism and later mafic intrusions.

Granodiorite is found as small, massive to moderately foliated plutonic bodies at SE G. El-Faraid group that intrude the surrounding tonalite rocks. The outcrops are generally medium- to coarse-grained, with a grey to dark grey color on fresh surfaces (Fig. 3f) and light grey to slightly brownish on weathered surfaces (Fig. 3g). The granodiorite is typically jointed, which locally controls block disintegration and spheroidal weathering. Xenoliths of mafic to intermediate composition are locally abundant, occurring as rounded to subangular enclaves, which are variably assimilated and range in size from a few centimeters to nearly one meter. These enclaves are commonly fine-grained and suggest mingling or mixing during emplacement. Pegmatitic and aplitic dykes cut the granodiorite, occurring as cross-cutting veins and irregular pods, particularly along joints and fractures (Fig. 3g).

**Fig. 4:**
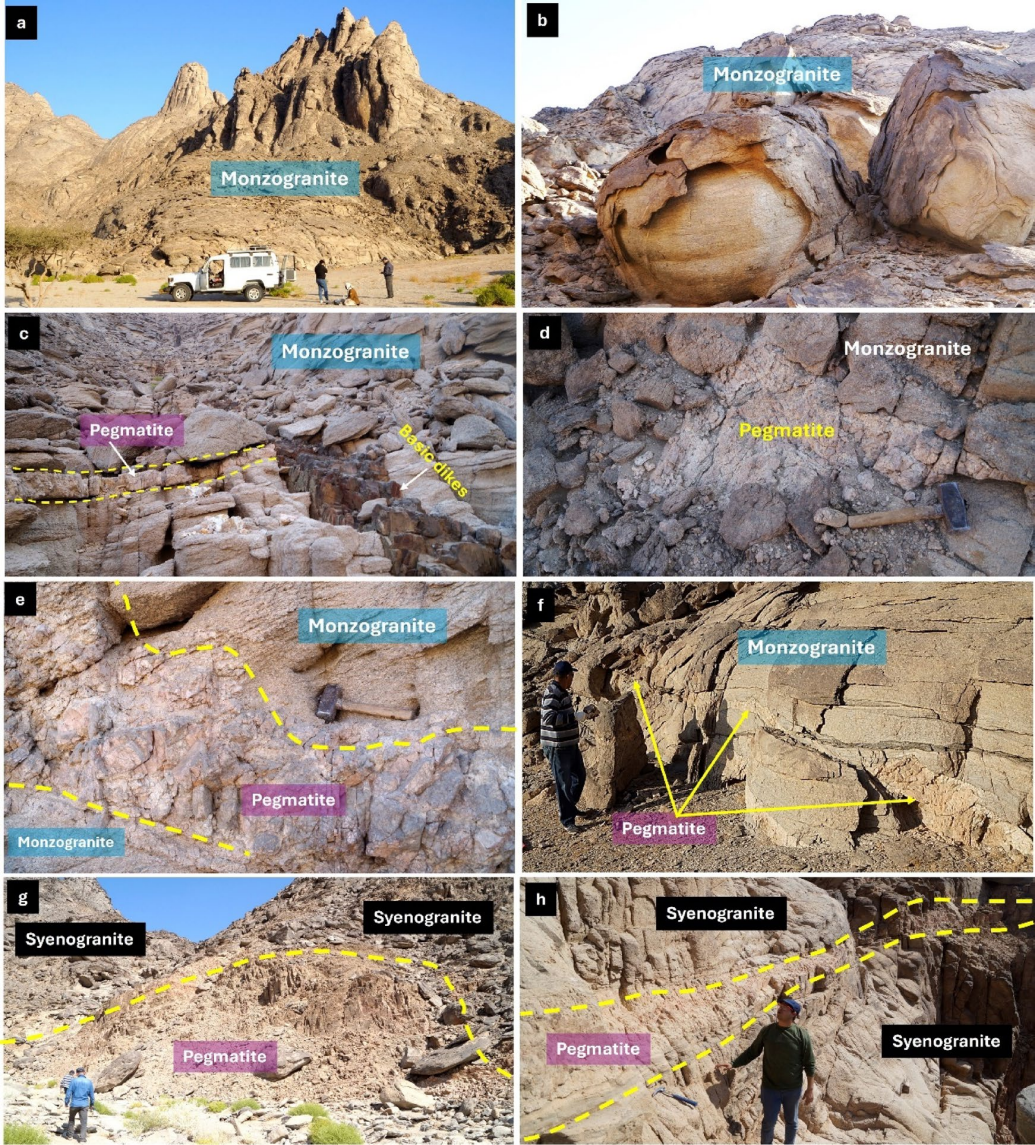
Field photographs at G. El-Faraid showing **a**) panorama view of G. El-Fereyid monzogranite, **b**) exfoliation in monzogranite, **c**) basic and pegmatitic dikes cutting each other, **d**) pegmatite pockets within monzogranite, **e**-**f**) pegmatite dike within Monzogranite at G. Marafai, and G. El-Fereyid respectively, **g**) pegmatite pockets within syenogranite, and **h**) pegmatite dike within syenogranite. All photographs were taken by the authors during field investigations. Written informed consent for publication was obtained from all identifiable individuals.

Monzogranite represents the dominant granitic rock found in G. El-Fereyid (south of the G. El-Faraid group), appearing in NW-SE elongated large plutonic bodies, lenses, and dykes intruded within tonalite (Fig. 4a). The monzogranite in G. El-Fereyid is characterized by a pinkish grey color, medium to coarse-grained texture, massive structure, and exfoliation (Fig. 4b). There are several fine-grained dykes and quartz veins cutting through the monzogranite rocks (Fig. 4c), which suggests that there was magmatic activity occurring after the formation of the monzogranite rocks. The dykes are mainly of both basic and acidic rocks, trending NE-SW (Fig. 4c). There are also granitic pegmatite rocks in G. El-Fereyid cutting through monzogranites (Fig. 4c-f). Monzogranites are also found as small plutons in G. El Marafai and G. Budkin.

Syenogranite represents one of the late-to post-tectonic intrusive phases ~ 620 − 580 Ma)^46^ within the.

G. El-Faraid Group. It typically occurs as medium- to large-sized plutons and subordinate elongated bodies intruding the older diorite-tonalite assemblage and granodiorite (Fig. 4e). In the field, syenogranite is easily recognized by its pink to reddish color, coarse-grained texture, and massive, relatively undeformed character. The rock is leucocratic and exhibits a hypidiomorphic granular fabric. Contacts with the host rocks are generally sharp to gradational and are commonly marked by a narrow thermal metamorphic aureole. Syenogranite bodies frequently exhibit exfoliation joints and are moderately weathered (Fig. 4f). They are intruded by quartz veins, pegmatitic pockets (Fig. 4c), indicating a continuation of magmatic and hydrothermal activity after emplacement.

Granitic pegmatite is widespread in the study area, occurring mainly as vein-type bodies (granitic and perthitic pegmatites) that are irregularly distributed in the northwestern and southern parts of G. El-Fereyid, as well as in the southwestern part of G. Marafai. The veins, pocket-like pegmatitic bodies are observed; they exhibit well-defined internal zoning and range in diameter from ~ 0.6 m to several meters. Zoning is characterized by feldspar-rich domains, indicative of progressive crystallization and magmatic differentiation (Fig. 4e). The vein-like bodies vary considerably in size, ranging from a few meters up to ~ 25 m in length, with widths generally less than 7 m (Fig. 4f). These pegmatites are typically very coarse-grained and display a wide range of colors, from reddish and buff to whitish and grayish black (Fig. 4g, h).

Three pegmatite types have been defined based on their mineralogy and morphology. The first type of pegmatite is less abundant and found as an irregular body in the southern part of the area. This muscovite-rich pegmatite is often very coarse-grained and contains large individual crystals of its common constituents. The second type of pegmatite is found in the northwestern part of the mapped area, dissecting the monzogranite, and it shows a NW-SE strike with variable dips. The third type is in the northern part of the mapped area, dissecting the tonalites and striking E-W.

## Methodology

### Remote sensing datasets

A multi-source remote sensing dataset was compiled to support lithological mapping, hydrothermal alteration delineation, structural lineament extraction, and mineral prospectivity assessment across the G. El-Faraid area. The datasets include multispectral optical data (Landsat-8 OLI, ASTER, Sentinel-2), hyperspectral imagery (PRISMA), and synthetic aperture radar (ALOS PALSAR). The selection of multiple sensors was deliberate: optical data provide spectral discrimination of surface mineralogy and lithology, radar data capture surface roughness and structural fabric independent of solar illumination and cloud cover, and hyperspectral data enable diagnostic identification of alteration mineral assemblages. These complementary information sources were integrated to overcome the limitations inherent to any single sensor.

***Landsat-8 OLI*** data were acquired from the USGS Earth Explorer portal (https://earthexplorer.usgs.gov/). The Landsat-8 OLI sensor provides 11 spectral bands spanning 0.43–12.51 μm, with a spatial resolution of 30 m for the multispectral bands and 15 m for the panchromatic band, and a temporal resolution of 16 days. The data were delivered as Collection 2, Level-2 surface reflectance products, which have been subjected to standard USGS atmospheric correction using the Land Surface Reflectance Code (LaSRC). Landsat-8 data were used for broad lithological discrimination, principal component analysis (PCA), band-ratio enhancement, and visualization of structural features using image composites.

***ASTER (Advanced Spaceborne Thermal Emission and Reflection Radiometer)*** data were obtained from the NASA Earthdata portal. ASTER comprises 14 spectral bands: three in the VNIR (15 m spatial resolution), six in the SWIR (30 m), and five in the thermal infrared (90 m). Level-1T terrain-corrected data were used, atmospherically corrected using the Fast Line-of-sight Atmospheric Analysis of Spectral Hypercubes (FLAASH) algorithm within ENVI. ASTER SWIR bands are particularly sensitive to hydroxyl, carbonate, and clay mineral absorption features and were used for PCA-based kinematic analysis and structural fabric visualization (AST984 false-color composite).

***Sentinel-2 MSI (Multispectral Instrument)*** data were downloaded from the Copernicus Open Access Hub (https://scihub.copernicus.eu/). The Sentinel-2 satellite provides 13 spectral bands spanning 0.44–2.19 μm. Spatial resolution varies by band: 10 m for bands B2, B3, B4, and B8 (VNIR), 20 m for bands B5, B6, B7, B8A, B11, and B12 (Red-Edge and SWIR), and 60 m for atmospheric bands B1, B9, and B10. Level-2 A surface reflectance products were used, which are atmospherically corrected using the Sen2Cor processor. Sentinel-2 data formed the primary optical input for machine learning-based lithological classification. Key band combinations used for visual lithological discrimination include an enhanced band-ratio composite (b11/b2, b11/b12, b11/b8×b4/b8 as RGB) and a decorrelation-stretch composite (b11, b8, b2 as RGB), which were used to guide training sample selection.

***PRISMA (PRecursore IperSpettrale della Missione Applicativa)*** hyperspectral data (scene ID: PRS_L2D_STD_20201002082501_20201002082505_0001) were acquired on 2 October 2020 and downloaded from the ASI PRISMA mission portal (www.prisma.asi.it). PRISMA is a push-broom imaging spectrometer with a spectral sampling interval of ≤ 12 nm, covering 66 bands in the VNIR (400–1010 nm) and 173 bands in the SWIR (920–2500 nm), with a 30 m spatial resolution and a 5 m panchromatic co-registered channel. Level-2D products which are atmospherically corrected using the ASI standard processing chain were used. Noisy and water vapor-affected bands in the SWIR (b42–b53, b86–b104, b156–b173) were identified and removed as a preprocessing step, retaining 176 usable bands for analysis. PRISMA data were used exclusively for hydrothermal alteration mineral index computation.

***ALOS PALSAR*** data (Granule ID: ALPSRP07509_FBD_F0450, acquired 21 October 2018 at 14:25:58 UTC) were obtained from the Earth and Remote Sensing Data Analysis Center (ERSDAC), Japan, via the Alaska Satellite Facility data portal (https://vertex.daac.asf.alaska.edu/). Fine mode dual-polarization (FBD; HH + HV) Level-1.5 data were used, with a 12.5 m pixel spacing, 16 bits per pixel, an observation swath of 83 km (range) × 81 km (azimuth), an incidence angle of 38.7°, and an off-nadir angle of 34.3°. ALOS PALSAR data were used for automated structural lineament extraction and, following co-registration with Sentinel-2, were combined with optical data for integrated lithological classification.

### Preprocessing and data harmonization

#### Geometric correction and co-registration

All datasets were projected to the WGS 1984 UTM Zone 36 N coordinate system. Landsat-8 Level-2 and Sentinel-2 Level-2 A products are delivered with geometric terrain correction applied, with sub-pixel georeferencing accuracy. ASTER Level-1T data are terrain-corrected but were re-registered to the Sentinel-2 reference image using a ground control point (GCP)-based approach, achieving a root mean square error (RMSE) of less than 0.5 pixels.

ALOS PALSAR Level-1.5 SAR data, although supplied as geo-referenced and geo-coded products, are known to carry inherent positional offsets arising from tropospheric delay, orbital uncertainty, and terrain-induced distortions. To mitigate this, a rigorous image-to-image co-registration was performed between the HH-polarization PALSAR scene and the Sentinel-2 reference image using a polynomial warping function of second order, with GCPs distributed uniformly across the scene to capture local terrain effects. Co-registration accuracy was verified against stable geological features (ridge crests and wadi confluences) visible in both datasets, achieving a final RMSE of less than one pixel (< 12.5 m).

#### Radiometric and atmospheric correction

Sentinel-2 Level-2 A and Landsat-8 Level-2 products were used as delivered, having been subjected to radiometric calibration and atmospheric correction by the data providers prior to distribution. ASTER data were atmospherically corrected using the FLAASH (Fast Line-of-sight Atmospheric Analysis of Spectral Hypercubes) module in ENVI 5.6. PRISMA Level-2D data are provided as atmospherically corrected surface reflectance and were used without further atmospheric processing, in accordance with ASI recommendations for geological applications. For ALOS PALSAR SAR data, radiometric terrain correction was applied using the Digital Elevation Model (DEM) from the Shuttle Radar Topography Mission (SRTM, 30 m resolution) to normalize backscatter intensity for local incidence angle variations and to reduce terrain-induced radiometric distortions.

#### Spatial resolution harmonization

The five datasets used in this study have differing native spatial resolutions: Sentinel-2 (10/20 m), ASTER VNIR +SWIR (15–30 m), PALSAR (12.5 m), PRISMA (30 m), and Landsat-8 (15–30 m). To enable spatial integration, Sentinel-2, ALOS PALSAR, and PRISMA datasets intended for machine learning classification and fuzzy-logic overlay analysis were resampled to a common grid of 10 m, selected as the finest common resolution consistent with the spatial fidelity of all contributing datasets, using a nearest-neighbor resampling algorithm. All resampling and reprojection operations were performed within ENVI 5.6 and ArcGIS Desktop 10.8.

### Image enhancement and multispectral analysis

Landsat-8 OLI data were processed using band-ratio techniques, decorrelation stretch composites, and principal component analysis (PCA) to enhance lithological contrast and highlight major structural features. For ASTER data, a false-color composite using bands 9, 8, and 4 (AST984) in RGB was generated to enhance spectral contrasts related to ferric iron and hydroxyl-bearing minerals. PCA was applied to the stacked ALOS PALSAR HH and HV polarization bands; the PC1 image derived from the stacked (HH + HV) and (HH − HV) band combination was used for structural fabric visualization and kinematic indicator identification. The PC1 composite was displayed in greyscale and in a false-color combination to highlight sigmoidal vein geometries and shear sense indicators.

Sentinel-2 data were processed through Minimum Noise Fraction (MNF) transformation prior to classification to reduce dimensionality, suppress noise, and concentrate geological information into fewer components. MNF-transformed Sentinel-2 components were used as the optical classification input (S2-MNF dataset). For the integrated classification, MNF-transformed Sentinel-2 bands were stacked with the co-registered ALOS PALSAR HH and HV polarization backscatter bands to produce the combined S2 + ALOS dataset.

### Structural lineament extraction

Automated lineament extraction was performed on the PC1 image derived from stacked ALOS PALSAR HH + HV data using the LINE algorithm in PCI Geomatica v.18. The LINE algorithm detects linear features through a three-stage process: edge detection using a Canny-type gradient filter, binary thresholding, and curve extraction via curve-linking with user-defined constraints. The parameter values used in this study are given in Table 1. These values were selected iteratively through sensitivity testing against field-measured structural orientations, and deviate in several parameters from program defaults to suppress false lineaments arising from wadi channels and alluvial surfaces while retaining geologically meaningful fracture traces. Lineament density was calculated using a kernel density estimation approach in ArcGIS Desktop 10.8 and used as a structural evidence layer in prospectivity mapping.

**Table 1:** Parameters used in the lineament extraction algorithm.

LINE Parameter	Value
Filter radius (RADI)	10
Edge gradient threshold (GTHR)	80
Curve length threshold (LTHR)	30
Line fitting threshold (FTHR)	3
Linking distance (DTHR)	20
Angular difference threshold (ATHR)	15°

### Hyperspectral alteration mapping

Six spectral mineral indices sensitive to hydrothermal alteration assemblages were computed from PRISMA Level-2D surface reflectance data: argillic alteration (alunite, kaolinite, pyrophyllite), clay minerals (smectite, illite), ferrous silicates (chlorite, epidote, biotite), ferrugination (hematite, goethite), hydroxyl-bearing minerals, and phyllic alteration (sericite, muscovite). Each index was formulated as a band ratio or arithmetic combination of PRISMA bands targeting diagnostic absorption features in the SWIR (1900–2500 nm) and VNIR (400–1000 nm) ranges. Output index maps were normalized to a 0–1 scale, classified into high-to-low intensity intervals, and subsequently used as evidential layers in the fuzzy-logic prospectivity analysis.

### Machine learning-based lithological classification

Two supervised machine learning classifiers Support Vector Machine (SVM) and Random Forest (RF) were applied to the S2-MNF and S2 + ALOS datasets to produce automated lithological maps for six target classes: metavolcanics (MV), metagabbro-diorite (Mgb), tonalite-granodiorite (TG), monzogranite (MGr), syenogranite (SG), and wadi deposits (WD). Training and validation samples were delineated based on existing geological mapping and visual interpretation of enhanced Sentinel-2 composites, with datasets split 80%/20% for training and validation respectively. SVM was implemented with a radial basis function (RBF) kernel, with regularization parameter C and kernel width γ optimized through a grid-search cross-validation approach. RF was configured with 100 decision trees, with node splitting governed by the Gini impurity criterion. Classification accuracy was evaluated using overall accuracy (OA), Kappa coefficient, and class-wise precision, recall, and F1-score derived from confusion matrices. All classification workflows were executed in ENVI 5.6.

### Mineral prospectivity mapping

A fuzzy-logic overlay was implemented in ArcGIS Desktop 10.8 to integrate three standardized evidential layers: PRISMA-derived alteration indices, ALOS PALSAR lineament density, and the SVM S2 + ALOS lithological classification map. Each layer was transformed to a continuous fuzzy membership scale (0–1) using appropriate membership functions (logistic for continuous alteration indices; small-function for distance-based structural proximity). The fuzzy gamma operator was applied to combine the standardized layers, with the gamma parameter (γ) optimized through iterative comparison against known mineralization indicators in the area. The resulting prospectivity map was classified into five favorability categories: very high, high, moderate, low, and very low.

### Field structural data collection and petrographic analysis

Fieldwork focused on systematic collection of structural data including foliations, lineations, fold orientations, shear zones, faults, and joints across all major lithological units. Kinematic indicators including σ- and δ-type porphyroclasts, S-C fabrics, asymmetric folds, boudinage, and quartz vein geometries were documented to establish shear sense and constrain the relative sequence of deformation events. Structural data were plotted and analyzed using Rockworks v.18. Petrographic analysis was conducted on 31 polished thin sections representing all major lithological units, examined under plane-polarized light (PPL) and cross-polarized light (XPL) using a polarized optical microscope at the Nuclear Materials Authority, Cairo, Egypt. Petrographic observations were correlated with remote sensing outputs to validate lithological assignments and constrain deformation timing.

## Machine learning-based lithological mapping

### Pre-classification image preparation and classifier background

#### Sentinel-2 band selection and MNF transformation

Prior to classification, Sentinel-2 MSI data were prepared through a systematic band selection and dimensionality reduction workflow. The ten multispectral bands with 10 m and 20 m nominal spatial resolutions B2 (490 nm), B3 (560 nm), B4 (665 nm), B5 (705 nm), B6 (740 nm), B7 (783 nm), B8A (865 nm), B11 (1610 nm), and B12 (2190 nm) were selected for processing. The three 60 m atmospheric correction bands (B1, B9, B10) were excluded, as they do not carry lithological information relevant to basement terrain mapping. All ten selected bands were resampled to a uniform 10 m pixel size using bilinear interpolation within ENVI 5.6 prior to further processing. Minimum Noise Fraction (MNF) transformation was applied to the ten-band Sentinel-2 stack to segregate data variance from noise, reduce data dimensionality, and concentrate geological spectral information into a smaller number of components. This MNF-transformed ten-component dataset is hereafter referred to as S2-MNF throughout the classification analyses.

#### Band-ratio composite and decorrelation stretch

**Fig. 5:**
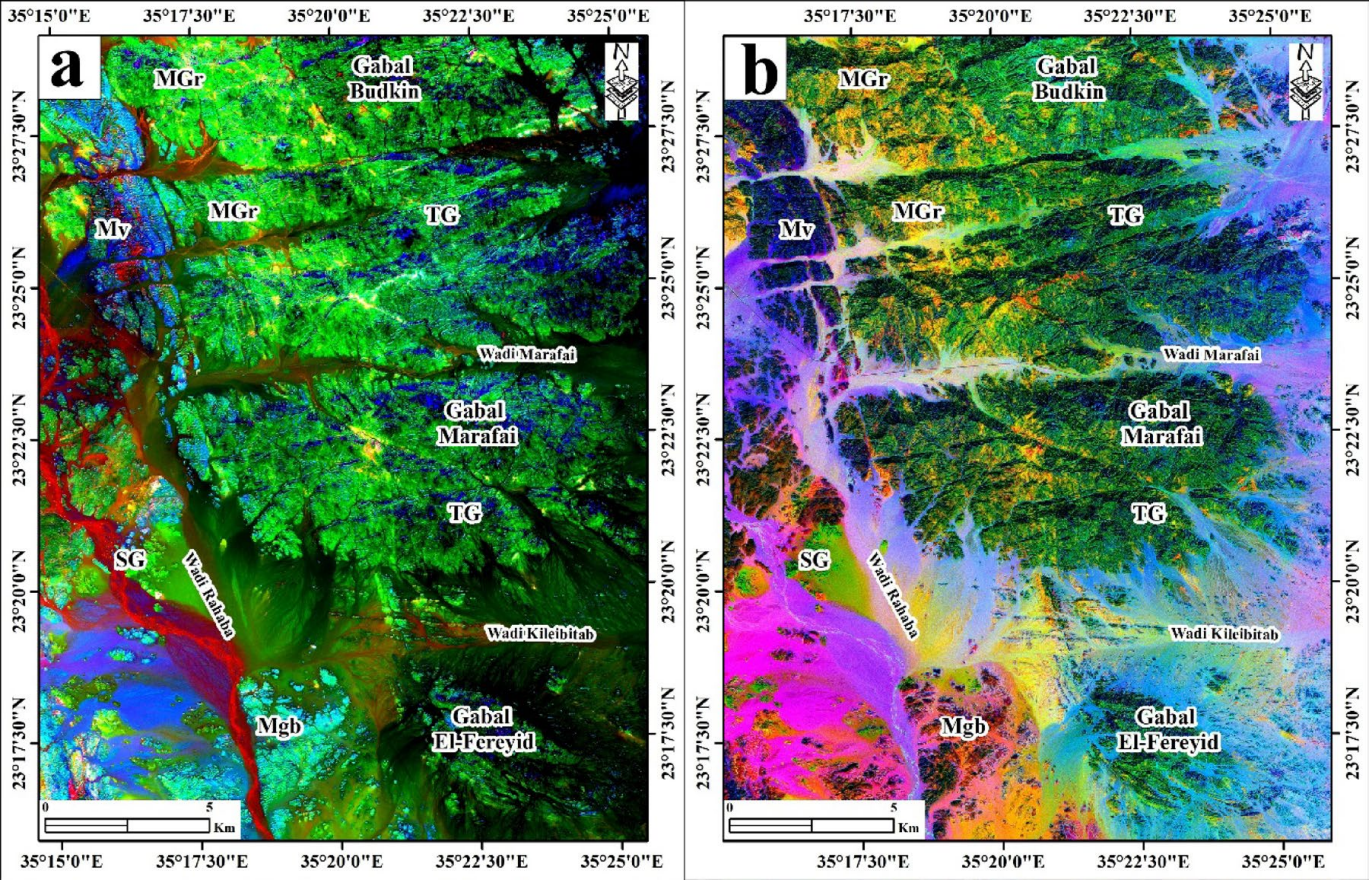
Lithological discrimination (**a**) using enhanced S2-band ratios (b11/b2, b11/b12, b11/b8×b4/b8) on RGB and (**b**) using S2-decorrelation stretch (b11, b8, b2) on RGB. Created by ENVI v. 5.3 software; https://www.l3harrisgeospatial.com/Software-Technology/ENVI, which is mainly utilized for image processing and ArcGIS Desktop 10.8. https://www.esri.com/en-us/arcgis/products/arcgis-desktop/overview.

To support training sample selection and provide an independent visual reference for lithological discrimination, two image enhancement composites were generated from the original Sentinel-2 surface reflectance bands. The first is an enhanced band-ratio composite, displayed as RGB using the following ratios: R = B11/B2, G = B11/B12, B = (B11/B8) × (B4/B8) (Fig. 5a). These ratios were designed to exploit specific spectral contrasts: the B11/B2 ratio (SWIR/Blue) enhances iron-bearing and clay-altered rocks against unaltered felsic lithologies; the B11/B12 ratio differentiates hydroxyl-bearing minerals (clays, micas) from carbonate and iron oxide phases through contrasting SWIR absorption slopes; and the (B11/B8) × (B4/B8) ratio amplifies ferric iron content by combining SWIR brightness with the visible-to-NIR spectral slope, thereby enhancing discrimination between mafic lithologies (metagabbro-diorite, metavolcanics) and felsic granitoids. Together, these ratios produce a composite (Fig. 5a) in which major lithological units are rendered in strongly contrasting colors, enabling reliable visual assignment of training pixels.

The second is a decorrelation stretch composite, displayed as RGB using Sentinel-2 bands B11 (SWIR-1), B8 (NIR), and B2 (Blue) (Fig. 5b). Decorrelation stretch is a statistical enhancement technique that removes the high inter-band correlation characteristic of multispectral imagery by transforming the data into principal component space, applying contrast stretching independently to each component, and transforming back to the original band space. The result is an image in which color saturation is greatly enhanced relative to a standard false-color composite, making subtle spectral differences between lithological units visually apparent. In the decorrelation-stretched B11-B8-B2 composite (Fig. 5b), tonalite-granodiorite, monzogranite, syenogranite, and metavolcanic units are rendered in distinct, highly saturated hues, facilitating boundary delineation between spectrally similar granitoid classes.

####  Integrated S2 + ALOS dataset

The integrated classification dataset, referred to throughout as S2 + ALOS, was constructed by stacking the ten retained S2-MNF components with the two co-registered ALOS PALSAR polarization backscatter bands: HH (horizontal transmit, horizontal receive) and HV (horizontal transmit, vertical receive). This produced a 12-band input feature space for classification. The inclusion of ALOS L-band SAR backscatter was driven by its sensitivity to surface roughness and dielectric properties that are not captured in optical reflectance data, providing complementary textural and structural information particularly useful for discriminating lithological units that share overlapping VNIR–SWIR spectral signatures^48^,^49^. Prior to stacking, all bands were co-registered to a common 10 m grid and normalized to a consistent dynamic range to prevent any single data source from dominating the classification feature space through scale effects.

#### Classifier descriptions

Support Vector Machine (SVM) is a supervised, kernel-based classifier that identifies optimal decision boundaries hyperplanes in a high-dimensional feature space by maximizing the margin between classes. For non-linearly separable data, SVM employs kernel functions to implicitly transform the input feature space into a higher-dimensional space where linear separation is achievable. In this study, a Radial Basis Function (RBF) kernel was applied, with the regularization parameter C and kernel width γ optimized through a five-fold cross-validation grid search. SVM is particularly well-suited to geological remote sensing classification tasks where training samples are limited relative to the number of spectral features and class boundaries are complex and nonlinear.

Random Forest (RF) is a supervised ensemble classifier that constructs a large number of decision trees during training, each built from a bootstrap sample of the training data and using a random subset of input features at each split. Final class predictions are determined by majority vote across all trees, which reduces the variance and overfitting associated with individual decision trees. RF is robust to spectral noise and can capture complex, non-Gaussian class distributions arising from intra-class mineralogical variability. In this study, RF was configured with 100 decision trees, with node impurity evaluated using the Gini criterion.

### Classification performance

**Fig. 6:**
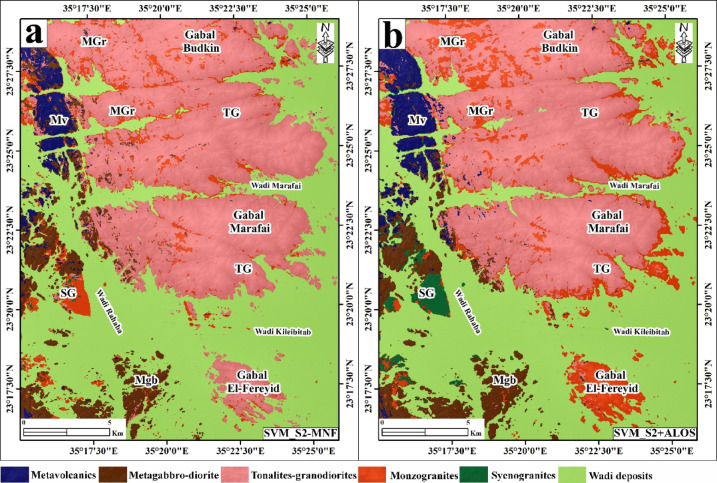
Lithological classification map generated using the Random Forest (RF) algorithm applied to the enhanced Minimum Noise Fraction (MNF) transformed Sentinel-2 data (**a**) and stacked Sentinel-2 and ALOS PALSAR data (**b**). Created by QGIS Desktop 3.36.3 software; https://qgis.org/project/visual-changelogs/visualchangelog336/ and ArcGIS Desktop 10.8. https://www.esri.com/en-us/arcgis/products/arcgis-desktop/overview.

**Fig. 7:**
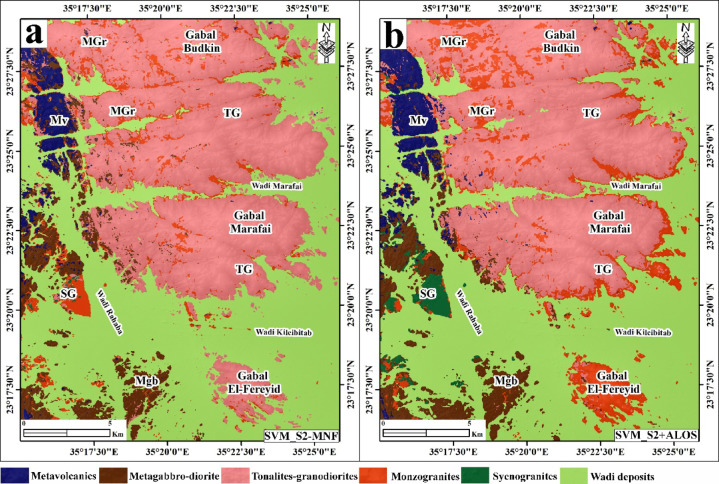
Lithological classification map generated using Support Vector Machine (SVM) algorithm applied to the enhanced Minimum Noise Fraction (MNF) transformed Sentinel-2 data (**a**) and stacked Sentinel-2 and ALOS PALSAR data (**b**). Created by QGIS Desktop 3.36.3 software; https://qgis.org/project/visual-changelogs/visualchangelog336/ and ArcGIS Desktop 10.8. https://www.esri.com/en-us/arcgis/products/arcgis-desktop/overview.

Both classifiers were applied independently to the S2-MNF and S2 + ALOS datasets, yielding four classification outputs. Training and validation datasets were constructed based on the existing geological map of the area^47^ and visual interpretation of the enhanced S2-MNF composites and band-ratio images described above. A total of 159,582 labeled pixels were used (S2-MNF) and 159,465 pixels (S2 + ALOS), divided 80% for training and 20% for validation across the six target classes: metavolcanics (MV), metagabbro-diorite (Mgb), tonalite-granodiorite (TG), monzogranite (MGr), syenogranite (SG), and wadi deposits (WD) (Table 2). Classification accuracy was evaluated using overall accuracy (OA), Kappa coefficient, and class-wise precision, recall, and F1-score derived from confusion matrices (Tables 3 and 4; Figs. 6 and 7).

The RF classifier applied to S2-MNF data yielded an OA of 89.58%, a Kappa coefficient of 0.8459%, and a mean F1-score of 85.63% (Fig. 6a; Table 3). Integration of ALOS SAR data improved RF performance to an OA of 91.43%, Kappa of 0.8727%, and mean F1-score of 87.51% (Fig. 6b; Table 3). SVM consistently outperformed RF across both data configurations. Using S2-MNF data, SVM attained an OA of 90.53%, Kappa of 86.16%, and mean F1-score of 86.20% (Fig. 7a; Table 4). The highest classification performance across all tested scenarios was achieved with SVM applied to the S2 + ALOS integrated dataset, yielding an OA of 92.67%, a Kappa coefficient of 89.28%, and a mean F1-score of 88.64% (Fig. 7b; Table 4).

**Table 2:** Training and testing data and abbreviations of the lithological classes.

Dataset	MNF-Sentinel-2			MNF-Sentinel-2 + ALOS PALSAR		
Lithological unit	Training pixels	Testing pixels	Sum	Training pixels	Testing pixels	Sum
Metavolcanic (MV)	7990	1997	9987	7986	1996	9982
Metagabbro-diorite (Mgb)	10,524	2631	13,155	10,502	2625	13,127
Tonalite-granodiorite (TG)	60,880	15,220	76,100	60,842	15,210	76,052
Monzogranite (MGr)	12,190	3047	15,237	12,187	3047	15,234
Syenogranite (SG)	1765	441	2206	1766	442	2208
Wadi deposits (WD)	34,318	8579	42,897	34,290	8572	42,862
Sum	127,666	31,916	159,582	127,572	31,893	159,465

**Table 3:** Confusion matrices, overall, and class-based statistics for the RF algorithm.

S2-MNF	MV	Mgb	TG	MGr	SG	WD	Sum	Precision%	Recall%	F1-score%
MV	7843	726	1341	65	12	0	9987	78.53	83.22	80.81
Mgb	570	12,370	67	146	2	0	13,155	94.03	86.41	90.06
TG	896	564	71,620	3002	18	0	76,100	94.11	90.8	92.43
MGr	35	572	5687	8867	7	69	15,237	58.19	60.73	59.43
SG	44	5	141	9	2007	0	2206	90.98	97.9	94.31
WD	36	79	22	2512	4	40,244	42,897	93.82	99.83	96.73
Sum	9424	14,316	78,878	14,601	2050	40,313	159,582	**OA = 89.58**		
Overall Accuracy%								**89.58**		
Kappa Accuracy%								**0.8459**		
Mean F1 Accuracy%								**85.63**		
S2 + ALOS	**MV**	**Mgb**	**TG**	**MGr**	**SG**	**WD**	**Sum**	**Precision%**	**Recall%**	**F1-score%**
MV	7840	623	1435	83	1	0	9982	78.54	86.96	82.54
Mgb	704	12,010	211	198	1	3	13,127	91.49	82.76	86.91
TG	398	1282	72,231	2129	11	1	76,052	94.98	91.11	93
MGr	23	484	4842	9865	1	19	15,234	64.76	77.29	70.47
SG	51	15	184	9	1949	0	2208	88.27	98.98	93.32
WD	0	97	380	479	6	41,900	42,862	97.76	99.95	98.84
Sum	9016	14,511	79,283	12,763	1969	41,923	159,465	**OA = 91.43**		
Overall Accuracy%								**91.43**		
Kappa Accuracy%								**0.8727**		
Mean F1 Accuracy%								**87.51**		

**Table 4:** Confusion matrices, overall, and class-based statistics for the SVM algorithm.

S2-MNF	MV	Mgb	TG	MGr	SG	WD	Sum	Precision%	Recall%	F1-score%
MV	7828	777	1291	39	52	0	9987	78.38	86.03	82.03
Mgb	566	12,399	57	118	1	14	13,155	94.25	85.15	89.47
TG	656	1014	70,006	4370	47	7	76,100	91.99	93.13	92.56
MGr	2	287	3578	11,287	4	79	15,237	74.08	65.49	69.52
SG	46	64	59	11	2022	4	2206	91.66	81.2	86.12
WD	1	21	178	1411	364	40,922	42,897	95.40	99.75	97.52
Sum	9099	14,562	75,169	17,236	2490	41,026	159,582	OA = 90.53		
Overall Accuracy%								90.53		
Kappa Accuracy%								0.8616		
Mean F1 Accuracy%								86.20		
S2 + ALOS	MV	Mgb	TG	MGr	SG	WD	Sum	Precision%	Recall%	F1-score%
MV	8530	594	747	111	0	0	9982	85.4500	84.36	84.91
Mgb	578	12,458	44	43	1	3	13,127	94.90	86.56	90.54
TG	800	545	71,277	3399	31	0	76,052	93.72	95.27	94.49
MGr	169	784	2642	11,351	169	119	15,234	74.51	72.47	73.47
SG	34	8	104	111	1950	1	2208	88.32	90.45	89.37
WD	0	3	0	649	5	42,205	42,862	98.47	99.71	99.08
Sum	10,111	14,392	74,814	15,664	2156	42,328	159,465	OA = 92.67		
Overall Accuracy%								92.67		
Kappa Accuracy%								0.8928		
Mean F1 Accuracy%								88.64		

### Classification accuracy and comparative analysis

Class-wise accuracy metrics and inter-algorithm comparisons across all four classification scenarios are summarized in Tables 3 and 4. Across all configurations, wadi deposits (WD) and tonalite-granodiorite (TG) achieved consistently the highest F1-scores, reaching up to 99.08% and 94.49% respectively with SVM applied to S2 + ALOS data. WD discrimination benefits from its spectrally distinctive surficial alluvial signature, which contrasts strongly with crystalline basement lithologies in both optical and SAR feature spaces. TG likewise exhibits sufficient VNIR–SWIR contrast from surrounding units to allow reliable separation under all tested conditions. Syenogranite (SG) and metagabbro-diorite (Mgb) showed moderate to high performance, with F1-scores ranging from 86.12% to 94.31% and 86.91% to 90.54% respectively. Metavolcanics (MV) achieved moderate F1-scores of 80.81–84.91%, reflecting partial spectral overlap with metagabbro-diorite arising from shared mafic mineral assemblages (amphibole, chlorite, epidote) that produce similar SWIR absorption features. The lowest and most variable performance was consistently observed for monzogranite (MGr), with F1-scores of 59.43–73.47% across all scenarios, attributable to overlapping quartz–feldspar–biotite assemblages shared with adjacent granitoid units and the additional spectral overprinting imposed by hydrothermal alteration and surface weathering.

SVM outperformed RF in overall accuracy across both data configurations. With S2-MNF, SVM achieved an OA of 90.53% versus 89.58% for RF; with S2 + ALOS, SVM reached 92.67% versus 91.43% for RF. Kappa coefficients followed the same trend, with SVM surpassing RF by up to 2.01% points. At the class level, the most pronounced inter-algorithm difference was observed for MGr, where SVM exceeded RF by 10.09% points in F1-score using S2-MNF data (69.52% vs. 59.43%), demonstrating that SVM’s kernel-based margin optimization is more effective at resolving subtle spectral distinctions between spectrally similar granitoid subclasses. Conversely, RF outperformed SVM for SG classification in both data configurations (94.31% vs. 86.12% with S2-MNF; 93.32% vs. 89.37% with S2 + ALOS), suggesting that the ensemble averaging of RF better captures the intra-class spectral variability characteristic of this unit.

Integration of ALOS PALSAR SAR data with Sentinel-2 MNF features consistently improved classification accuracy for both algorithms. For SVM, OA increased from 90.53% to 92.67% (+ 2.14% points), Kappa from 0.8616% to 0.8928% (+ 3.12 points), and mean F1-score from 86.20% to 88.64% (+ 2.44 points). The most significant class-level improvement was again observed for MGr, with F1-score gains of + 11.04% points under RF and + 3.95 points under SVM, confirming that L-band SAR backscatter provides complementary textural and structural information that is critical for discriminating Lithologically unclear granitoid units where optical reflectance alone is insufficient. Minor F1-score decreases were observed for Mgb and SG under RF when transitioning from S2-MNF to S2 + ALOS (− 3.15% and − 0.99% respectively), reflecting increased radar backscatter noise in areas where these units exhibit similar surface roughness characteristics. Overall, the SVM classifier applied to the S2 + ALOS integrated dataset yielded the highest mapping performance across all metrics (OA = 92.67%, Kappa = 89.28%, mean F1 = 88.64%) and was adopted as the basis for the final lithological map of the study area.

## Hydrothermal alteration mapping using PRISMA dataset

The spatial distribution of diverse hydrothermal alteration zones across the study area was delineated utilizing PRISMA hyperspectral data, yielding six distinct mineralogical and alteration indices (Fig. 5). The resulting maps display the relative intensity (High–Low) of argillic alteration, clay minerals, ferrous silicates, ferrugination, hydroxyl-bearing minerals, and phyllic alteration across a structurally complex terrain. The area is dominated by three main granitic massifs: Gabal Budkin in the north, Gabal Marafai in the central domain, and Gabal El-Fereyid in the south, dissected by a network of structurally controlled wadis including Wadi Rahaba, Wadi Marafai, and Wadi Kileibitab.

**Fig. 8:**
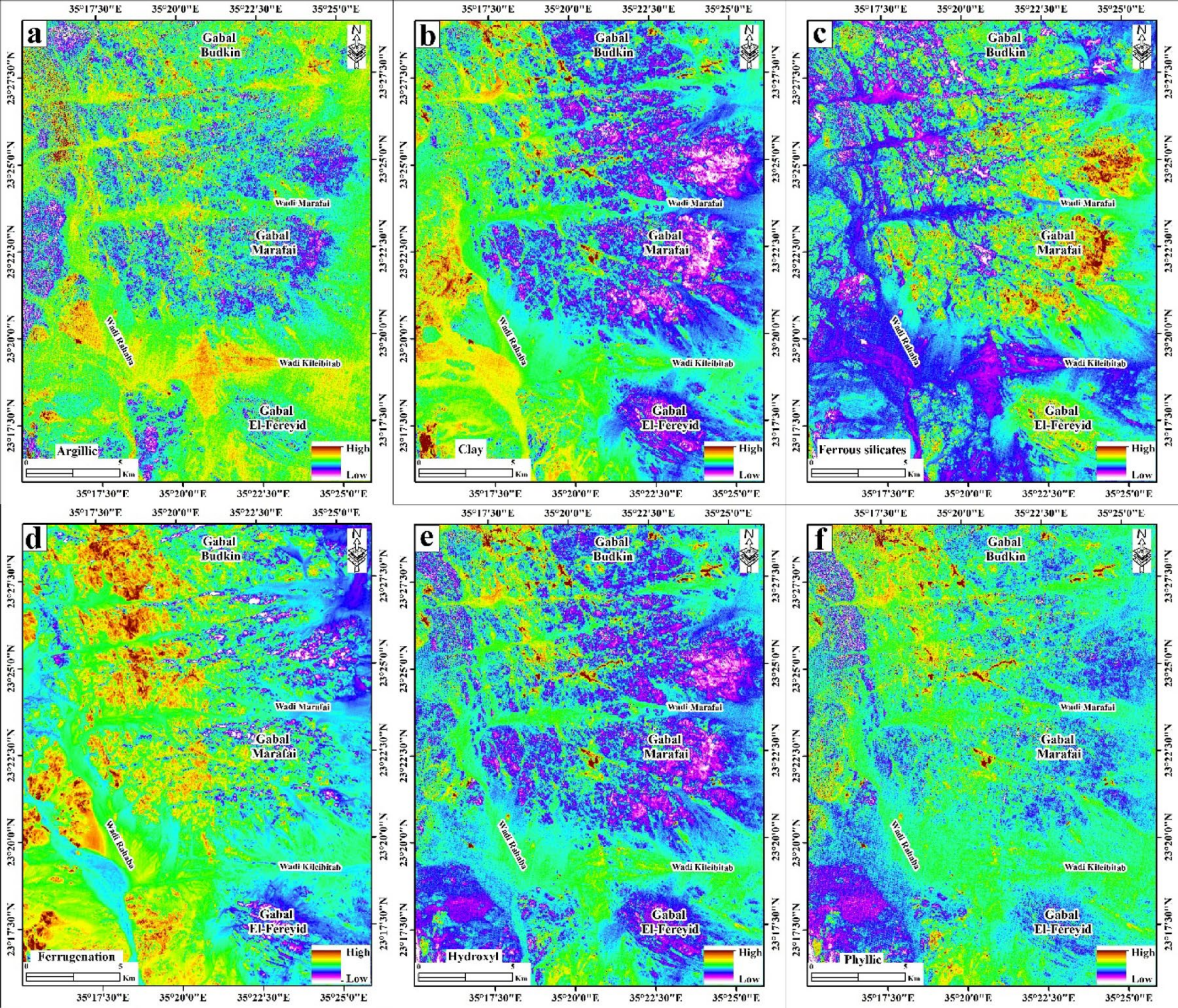
Hyperspectral PRISMA alteration and mineral indices in the G. (**a**) Argillic alteration index (b132 + b194/ b189), emphasizing alteration zones enriched in alunite, kaolinite, and pyrophyllite. (**b**) PRISMA index (b189×b201)/(b194 × b194) detects the clay alteration zones. (**c**) PRISMA index (b189/b132) emphasizing the ferrous silicate (biotite, chlorite, amphibole) alteration zones. (**d**) Ferrugination index (b132/b33 + b33/b21) emphasizes the distinct alteration zones rich in hematite and goethite. (**e**) PRISMA index (b201/b194) × (b132/b194) emphasizes the hydroxyl-bearing alteration zones. (**f**) PRISMA index (b189 + b201)/b194, highlighting phyllic alteration zones. Created by ENVI v. 5.3 software; https://www.l3harrisgeospatial.com/Software-Technology/ENVI, which is mainly utilized for image processing and ArcGIS Desktop 10.8. https://www.esri.com/en-us/arcgis/products/arcgis-desktop/overview.

Argillic alteration (Fig. 8a) exhibits diagnostic absorption near 2165 nm, was mapped using the PRISMA index (b132 + b194)/b189. It is concentrated mainly along the major wadis and in the peripheral zones of the granitic massifs, forming laterally continuous alteration belts. High argillic intensity is particularly evident along Wadi Marafai, where elongate, NW–SE to NNW–SSE trending anomalies follow the main drainage course and associated lineaments, and along Wadi Kileibitab, where discrete high-value patches mark structurally controlled zones cutting the southern flank of Gabal Marafai and the northern margin of Gabal El-Fereyid. Around Gabal Budkin and Gabal Marafai, argillaceous responses define discontinuous halos that are more pronounced on the down-slope, wadi-facing margins than in the interiors of the plutons, indicating well-developed proximal–distal gradients away from the intrusive cores. The clay-minerals index (b189×b201)/(b194 × b194) (Fig. 8b), as a type of argillic alteration, shows a broadly similar but more extensive pattern. A continuous, high-response clay band encircles much of Gabal Marafai, forming a well-defined alteration halo that joins with strong clay anomalies along Wadi Marafai and its northern tributaries.

In contrast, clay intensities around Gabal Budkin are more fragmented, occurring as isolated high-value clusters along its eastern and southern flanks, whereas Gabal El-Fereyid shows a comparatively subdued but spatially continuous clay halo that is elongated NW–SE in agreement with the mapped pluton geometry. Ferrous-silicate alteration (Fig. 8c), such as chlorite and epidote, marked by Fe-Mg-OH absorption features, was detected using the PRISMA index b189/b132. It is markedly concentrated within and immediately adjacent to the granitic bodies, especially Gabal Marafai and Gabal Budkin, where high-intensity anomalies form irregular but internally continuous cores. The strongest ferrous-silicate responses occur in the central and eastern parts of Gabal Marafai and in the southern segment of Gabal Budkin, locally extending into adjacent wadis as narrow tongues aligned with mapped fault and fracture trends.

Gabal El-Fereyid exhibits weaker, more patchy ferrous-silicate signatures, mainly restricted to its northern and northeastern margins, where they coincide with granitic country-rock contacts and known fracture corridors. Ferrugination index (b132/b33 + b33/b21) (Fig. 5d), representing iron oxide enrichment associated with hematite and goethite mineralization and characterized by absorption near 600 and 900 nm, is dominantly wadi-controlled, with high iron oxide intensity outlining the main drainage network and structurally disrupted zones. Along Wadi Marafai, ferrugination forms a broad, high-response corridor that parallels the wadi axis and locally merges with argillaceous and clay anomalies, indicating laterally continuous oxidation zones along fault-controlled fluid pathways. Around Gabal Budkin, ferruginous anomalies are concentrated on the western and southwestern slopes and along wadi intersections, while Gabal El-Fereyid shows discrete ferruginous patches along its western margin and downstream into Wadi Kileibitab, defining elongate alteration trains that follow the regional NW-SE structural grain.

Hydroxyl-bearing minerals index (b201/b194) × (b132/b194) (Fig. 8e) displays the most pervasive distribution, with extensive moderate-to-high responses encircling all three granitic massifs and infilling the major valleys. Around Gabal Marafai, a broad, high-intensity hydroxyl halo overlaps the clay-rich zones and extends distally along Wadi Marafai, producing a continuous alteration corridor from the pluton interior to the basin outlets. Gabal Budkin is fringed by discontinuous but comparatively intense hydroxyl anomalies, particularly on its southern and eastern flanks, whereas Gabal El-Fereyid is characterized by a more symmetric but lower-intensity halo, consistent with earlier mapping that documents pervasive but moderate alteration in its monzogranite and pegmatite systems.

Phyllic alteration index (b189 + b201)/b194 (Fig. 8f) is more restricted in extent but spatially focused along structurally controlled trends, forming narrow, high-intensity bands that commonly overprint clay and hydroxyl-rich zones. The most pronounced phyllic anomalies occur along the northern and northeastern margins of Gabal Marafai and the southern flank of Gabal Budkin, where they define NNE–SSW to NNW–SSE oriented belts coincident with major fault and fracture sets reported from the area. Additionally, smaller phyllic patches fringe the northern contact of Gabal El-Fereyid toward Gabal Marafai, delineating a structurally linked corridor between the two plutons that is also highlighted by elevated ferrous-silicate and hydroxyl responses.

Overall, the strong, spatially continuous anomalies in all six indices and the well-developed, multi-ring alteration halos that converge in Wadi Marafai and Gabal Marafai have the highest overall alteration intensity of the three major plutons. Anomalies of ferrous silicate, ferruginous, and hydroxyl are concentrated along structurally controlled slopes and wadis in Gabal Budkin, which exhibits high but more spatially discontinuous alteration. Although localized high-intensity anomalies coincide with structurally disturbed zones and pegmatite-bearing parts described in the geological investigation, Gabal El Fereyid records the lowest relative intensities, characterized by broad but weaker clay and hydroxyl halos, and limited ferrous silicate and phyllic development.

## Remote sensing-based structural analysis

### Automated lineament extraction

**Fig. 9:**
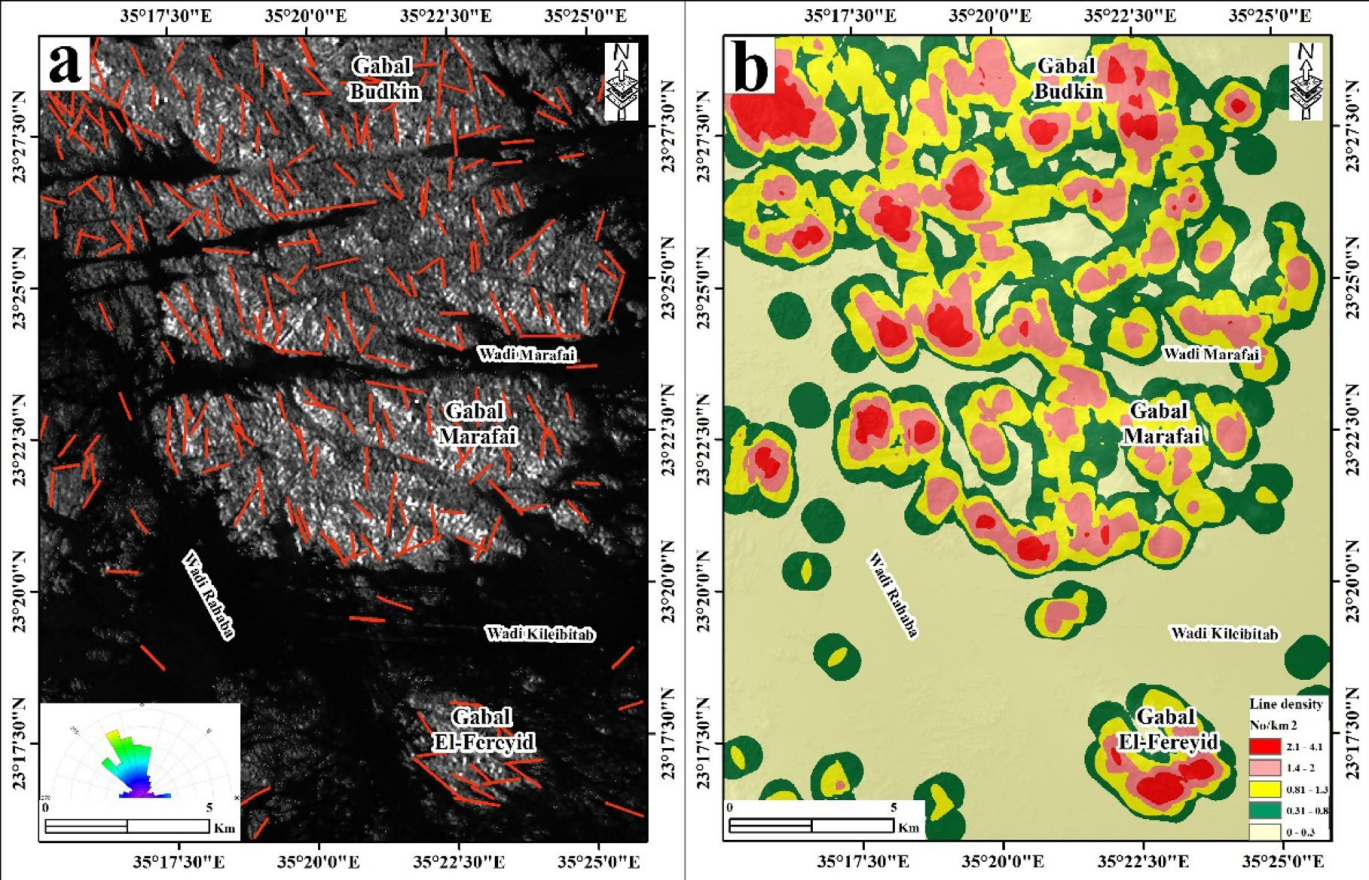
(**a**) Lineaments extraction and rose diagrams based on ALOS PALSAR data. (**b**) Lineament density map of ALOS PALSAR data. Created by ArcGIS Desktop 10.8. software; https://www.esri.com/en-us/arcgis/products/arcgis-desktop/overview, ENVI v. 5.3. software; https://www.l3harrisgeospatial.com/Software-Technology/ENVI, Geomatica PCI software, and RCOKWORK v. 18 software; https://www.rockware.com/product/rockworks/.

The automated lineament extraction over the Gabal Budkin, Gabal Marafai, and Gabal El-Fereyid area yields a dense and spatially heterogeneous fracture network characterized by well-defined structural corridors and pronounced orientation anisotropy (Fig. 9). Lineaments are distributed across the entire scene, but their geometry, spacing, and clustering vary systematically between the major plutons and surrounding wadis. In Fig. 9a, lineaments occur predominantly as straight to slightly curvilinear segments, forming fracture swarms of variable length, with individual traces locally coalescing into composite structural corridors spanning several kilometers. Lineament frequency is highest over the crystalline massifs of Gabal Budkin and Gabal Marafai, where closely spaced, subparallel sets define a strongly anisotropic fabric, whereas the intervening wadis and low-relief areas display more widely spaced and discontinuous lineaments. Southward, Gabal El-Fereyid is characterized by fewer but longer lineaments that delineate the main granitoid margins and internal fracture zones. The rose diagram (Fig. 9a) indicates two dominant orientations, a major NW–SE to NNW–SSE set and a subordinate NE–SW set, accompanied by minor E–W and N–S subsets. The NW–SE to NNW–SSE lineaments form the most laterally continuous structural corridors and account for the bulk of the mapped fracture length, whereas NE–SW lineaments locally intersect or truncate the former, producing orthogonal and rhombic fracture patterns. This bimodal orientation pattern confirms a pronounced directional diagonal in the fracture network and highlights the structural anisotropy of the study area. The lineament density map (Fig. 9b) further quantifies these patterns, with density values ranging from very low (0–0.3 lineaments/km²) to very high (2.1–4.1 lineaments/km²). Very high- and high-density maxima (≥ 1.4 lineaments/km²) form elongated belts that closely follow the crystalline cores of Gabal Budkin and Gabal Marafai, particularly in their central and northern sectors, where overlapping fracture swarms coalesce into continuous structural corridors. Moderate densities (0.81–1.3 lineaments/km²) outlying these cores and extend outward along the main lineament sets, whereas low to very-low densities dominate distal areas, especially in the broader wadi floors and peripheral sediment-covered zones.

Around Gabal El-Fereyid, density values are generally moderate to high, forming discrete clusters that mark localized fracture concentrations rather than a single continuous corridor. Collectively, Fig. 9 delineates several structurally significant zones characterized by high lineament frequency, strong orientation coherence, and well-organized fracture networks that partition the region into distinct structural domains, which play an important role in hydrothermal fluid migration and mineralization concentrations in the study area. The area is structurally controlled by NW-SW-trending structures and their conjugate NE-SW and N-S related to the Najd fault system, which affects mineralization in the Eastern Desert^4^,^5^,^19^,^24^,^32^,^52^.

### Kinematic indicator observation by remote sensing imaging processing

**Fig. 10:**
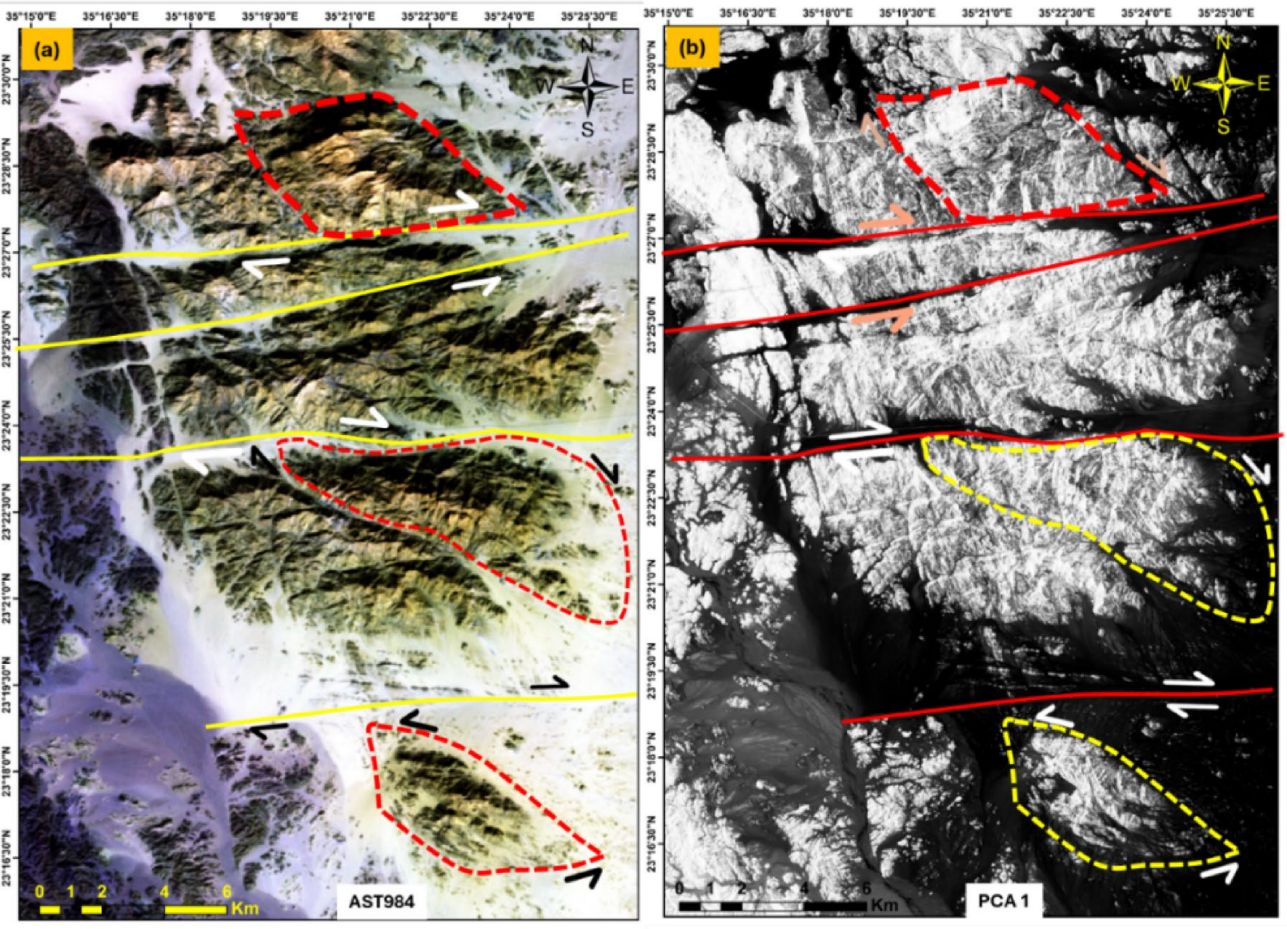
showing **a**) AST 984 FCC, and **b**) showing PC1 sinstral and dextral sense of shears, G. El-Faraid group. Created by ArcGIS Desktop 10.8. software; https://www.esri.com/en-us/arcgis/products/arcgis-desktop/overview, ENVI v. 5.3. software; https://www.l3harrisgeospatial.com/Software-Technology/ENVI, Geomatica PCI software, and RCOKWORK v. 18 software; https://www.rockware.com/product/rockworks/.

Kinematic indicators extracted from Landsat-8 OLI and ASTER reveal structural fabrics with uniform orientation and asymmetry, characteristic of ductile shear deformation. These indicators include sigmoidal veins observed in processed imagery using techniques such as PCA and band ratios (Fig. 10a, b).

The analysis highlights a dominant dextral and a few sinistral shear senses along major shear zones in the study areas. However, the observed shear indicators show near concordance with those reported from the ANS, possibly reflecting differences in regional tectonic stress regimes or temporal deformation phases. The shear sense indicators obtained from remote sensing in G. El-Faraid show complete harmony with field studies.

### Field structural observations and deformation events

Detailed field investigations reveal a polyphase deformation history, characterized by multiple generations of ductile and brittle structures, including foliations, lineations, folds, thrusts, strike-slip faults, dip-slip faults, and joint systems. These structures, including foliation developed in gneisses and granitoids, are mostly oriented NW-SE, parallel to the regional shear fabric of the ANS. Open to tight folds affect gneissic and tonalitic lithologies, with fold axes trending E-W to NE-SW. Brittle structures are widespread, particularly faults and fractures trending NW-SE and NE-SW. These structures of the G. El-Faraid area reflect a progressive tectonic evolution from early ductile contraction to late brittle deformation. The dominant structural grain is NW-SE, consistent with regional Pan-African shear directions within the southeastern ANS.

#### Contractional structures

**Fig. 11:**
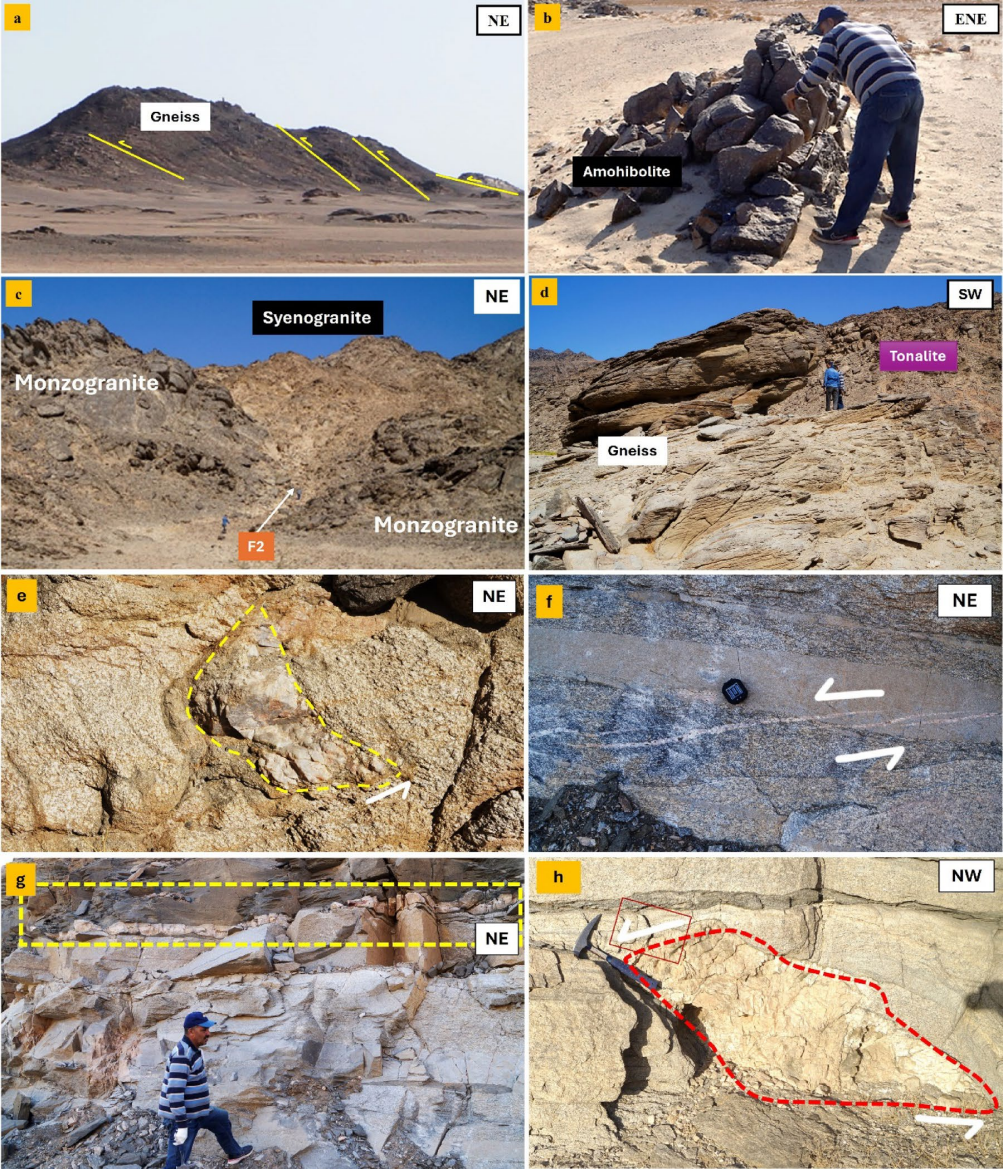
Field photograph at G. El-Faraid **a**) Typical thrusting at gneiss, **b**) Well-developed S1 foliation and L1 stretching lineation in foliated amphibolites driven by NNW-SSE, **c**) showing anticline-syncline fold within tonalite intruded near its core by synogranites, **d**) showing highly foliated and deformed gneiss, e) δ- type in tonalite showing dextral sense of shear with tonalite, **f**) a typically en echelon quartz veinlets with sinistral sense of shearing recorded with tonalite, **g**) deformed quartz vein veins within tonalite, and **h**) showing boudins within monzogranite indicate sinistral sense of shearing. All photographs were taken by the authors during field investigations. Written informed consent for publication was obtained from all identifiable individuals.

The earliest identifiable phase of deformation in the study area is responsible for the development of a pervasive S1 foliation and L1 stretching lineation in the metamorphic and ophiolitic units, including serpentinites, amphibolites, gneiss, and metavolcanics in the northwest portion of the G. El-Faraid near to Wadi El- Rahaba area, the S1 foliation is generally parallel to the primary bedding (S0), particularly well expressed in the volcaniclastic and gneiss (Fig. 11a). The exposed amphibolites in the G. El-Faraid area exhibit stretching lineation and prominent foliation, all of which are indicative of ductile deformation (the first deformation event (D1) (Fig. 11a, b). Their fabric and structural orientation show deformation during the contraction phase, associated with nappe transport and thrusting directed by the NNW-SSE and by early Pan-African arc accretion. As a result, these amphibolites document early crustal shortening and provide a mechanically anisotropic framework that later influenced the emplacement of both syn- and post-orogenic granitoids. Another important structural feature is the formation of a distinct crenulation cleavage (S2), which is axial-planar to a later generation of macroscopic to mesoscopic-scale folds (F2) (Fig. 11c).

A series of wide antiforms and synforms have formed as a result of F2 deformation, among which two are more pronounced. One such feature is the Faraid antiform, located in the central part of the study area, a large-scale asymmetric fold with a strike length exceeding 15 km. While the northeastern limb is moderately dipping at 25° to 30°, the southwestern limb is more steeply dipping at 40° to 50°. The central part of this antiform is occupied by late tectonic synogranite bodies, thus indicating their syn- to post-tectonic emplacement with respect to F2 folding. These observations indicate that the second deformational event (D2) is actually the regionally most widespread tectonic event within this area, effectively overprinting earlier structural features formed during D1 deformation.

#### Transcurrent structures

These structures are marked by the transition from ductile thrusting and folding to transpressional strike-slip faulting. It is expressed through the development of NE-SW and ENE-trending strike-slip shear zones, particularly the subsidiary faults in G. El-Faraid. These structures displace older fabrics and fold hinges, producing localized zones of high strain with well-developed mylonitic foliation and stretching lineations subparallel to shear zone trends, which correlated to the third deformational event (D3). Kinematic indicators such as rotated porphyroclasts and synthetic shear bands confirm a dominantly sinistral sense of motion (Fig. 11e, f). In G. El-Faraid, D3-related shear zones reorient earlier fold axes and segment older lithological contacts. Quartz veins and felsic dykes within these zones are commonly folded and boudinaged and asymmetrically deformed (Fig. 11e-g).

#### Brittle structures

**Fig. 12:**
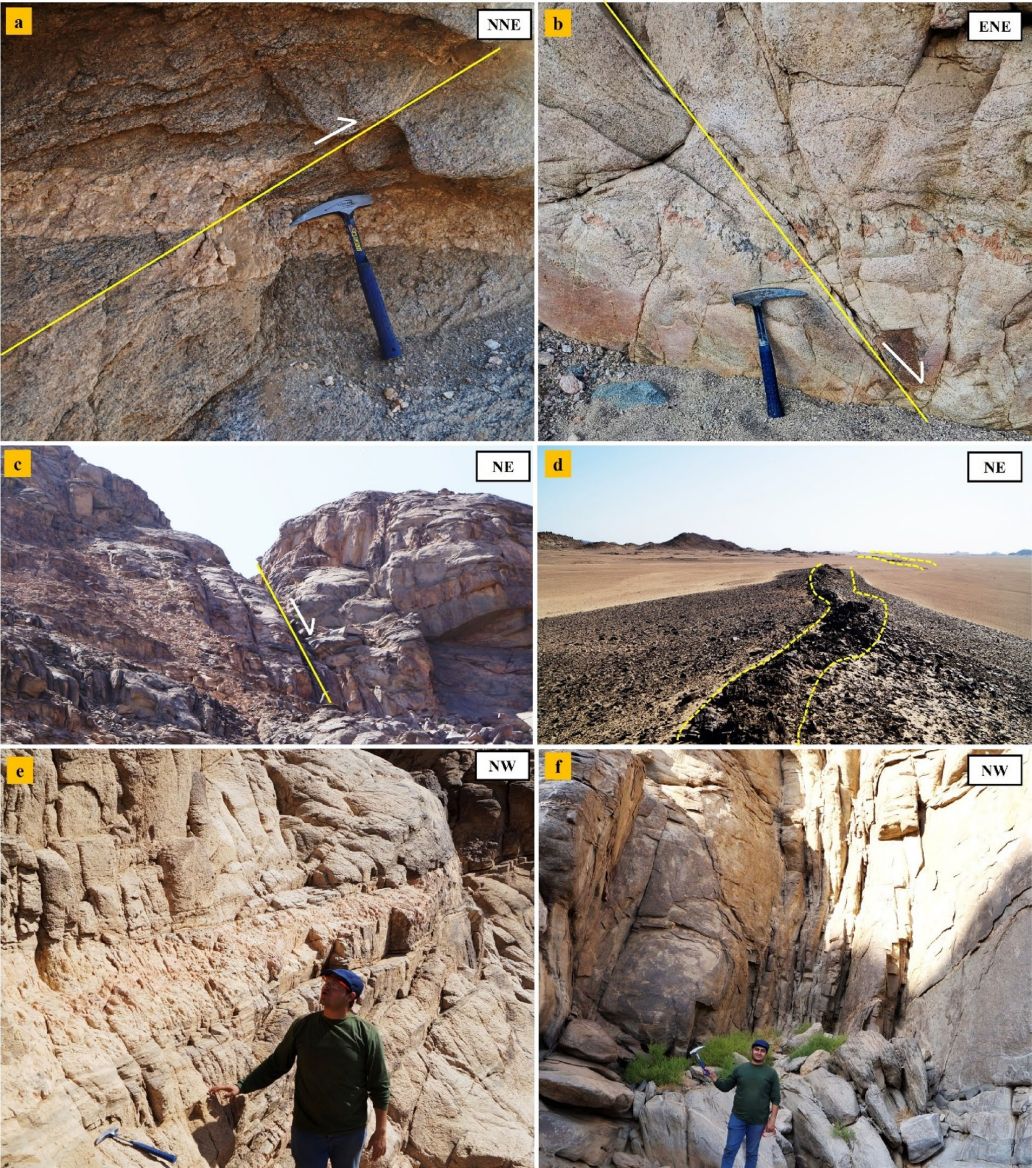
Field photographs at G. El-Faraid **(**a-c**)** Close-up view showing intersected reverse faults at the monzogranite southeast of G. El-Faraid, d) dextral strike-slip fault emphasized by movement of basic dike, e) showing two sets of joints cutting across the monzogranite, and f) Two joint sets in tonalite. All photographs were taken by the authors during field investigations. Written informed consent for publication was obtained from all identifiable individuals.

These structures are characterized by broad open warping and brittle faulting, likely associated with post-orogenic uplift and exhumation under shallow crustal conditions. Structures attributed to the fourth deformational event (D4) reflect the final stage of tectonic evolution, including brittle faults with minor displacements, both normal and strike-slip (Fig. 12a- d), and well-developed joint systems that cross-cut all earlier foliations and plutonic bodies trending NW-SE and NE-SW, typically steep to vertical (Fig. 12e, f). These brittle structures acted as conduits for late-stage hydrothermal fluids, controlling vein emplacement and alteration. These late-stage structures suggest a general relaxation of compressional stresses and the onset of an extensional to transtensional regime in the waning stages of the PAO^51^–^54^.

## Petrography

The G. El-Faraid Group is characterized by a diverse suite of late-to post-orogenic granitoids, representing an important segment of the northern ANS. Petrographically, the group comprises tonalite- granodiorite, monzogranite, syenogranite, and granitic pegmatites, which collectively record the magmatic and tectonic evolution of the area during the waning stages of the PAO.

**Fig. 13:**
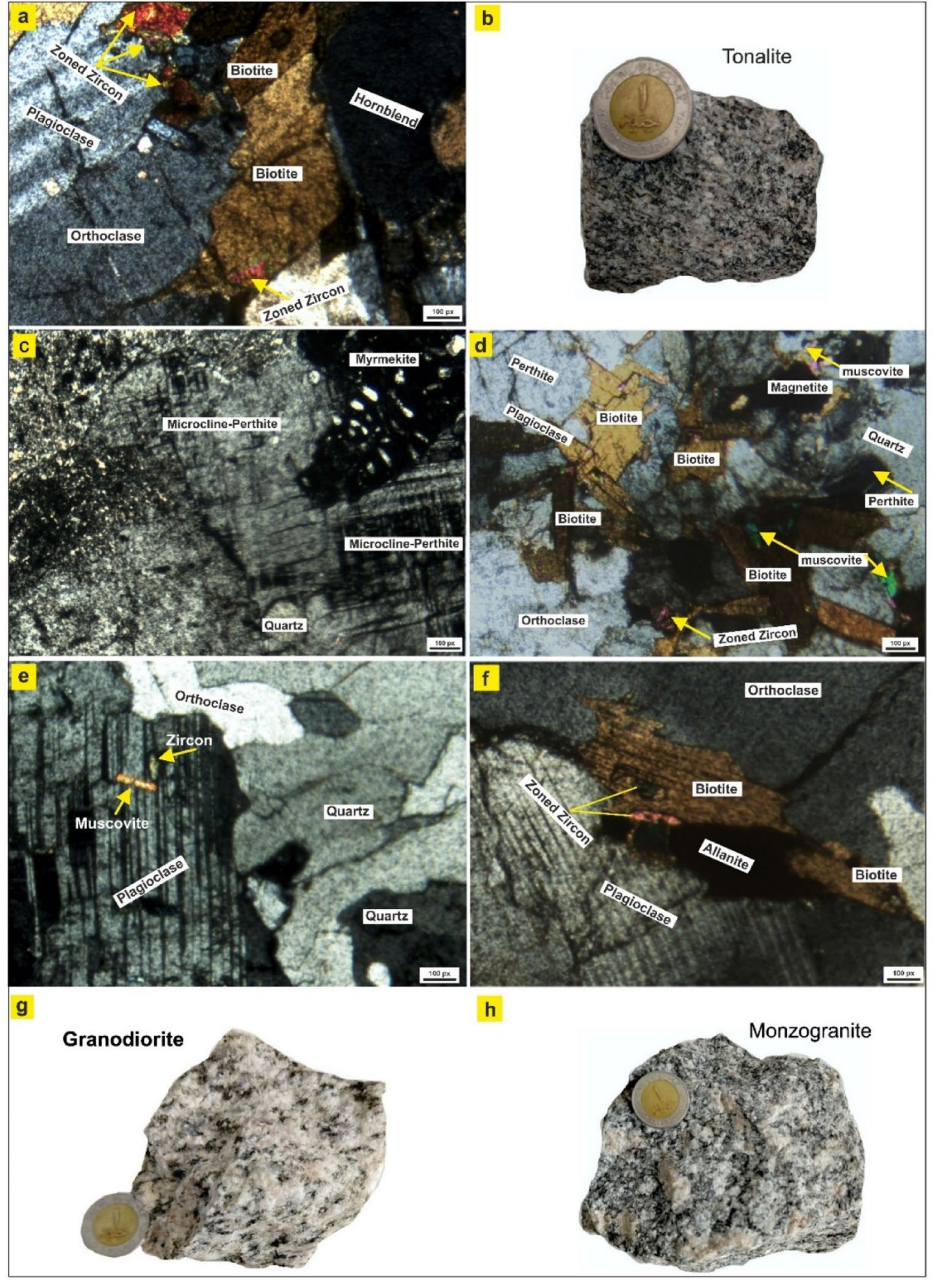
**a**) XPL photomicrograph showing plagioclase, orthoclase, biotite, hornblende, and abundant zoned zircon, **b**) Hand specimen of tonalite with medium- to coarse-grained, porphyritic texture, **c**) XPL view of microcline-perthite with associated quartz and myrmekitic intergrowths, **d**) XPL photomicrograph showing perthitic K-feldspar, plagioclase, quartz, biotite, muscovite, opaque minerals, and zoned zircon, e) Photomicrographs show plagioclase with polysynthetic twinning, quartz, orthoclase, and accessory zircon and muscovite, **f**) XPL photomicrograph showing plagioclase, orthoclase, biotite, allanite, and zoned zircon, g-h) Hand specimens of granodiorite and monzogranite, respectively, showing coarse-grained, holocrystalline textures. Figures 13 and 14 were taken by a polarized microscope at the Nuclear Material Authority, Cairo, Egypt (NMA).

Tonalite mainly contains plagioclase, lesser amounts of K-feldspar and quartz, and mafic minerals such as hornblende and biotite. Plagioclase occurs as anhedral to subhedral tabular crystals of oligoclase. It is characterized by lamellar twinning (Fig. 13a). Plagioclase crystals show variable degrees of saussuritization, while others are intensely fractured and fragmented, locally forming a mortar texture indicative of deformation-related cataclastic effects (Fig. 13c). K-feldspar (8–10 vol%) occurs as anhedral to subhedral crystals, commonly showing perthitic intergrowths (Fig. 12a-c), and is partially altered to clay minerals. Quartz occurs as anhedral to subhedral crystals interstitial to the early-crystallized phases (Fig. 13c). It commonly displays undulose extinction, with some cracks, and locally granulated because of brittle deformation and cataclastic overprinting (Fig. 12a-c).

Biotite mineral is subhedral, commonly fractured with pale brown colour and weak pleochroism from pale brown to very pale greenish brown. It is commonly altered to chlorite and iron oxides along cleavage planes and locally hosts zircon inclusions (Fig. 13a). Secondary muscovite occurs as very fine flakes associated with recrystallized and reworked quartz grains. Hornblende is present as a subhedral prismatic crystal showing strong pleochroism from pale green to green and yellowish-green (Fig. 13a). Accessory minerals comprise zircon, allanite, fluorapatite, and opaque phases, which are distributed within the rock and frequently associated with both felsic and mafic components (Fig. 13a-c). Zircon occurs as euhedral prismatic crystals showing well-developed oscillatory zoning, a diagnostic feature of magmatic growth during progressive crystallization of the tonalitic melt (Fig. 13a).

The granodiorite consists mainly of plagioclase, quartz, K-feldspar, biotite, and hornblende, with accessory zircon, titanite, apatite, and opaques. Plagioclase is the dominant feldspar (Pl 40-58.3 vol%), occurring as subhedral tabular crystals ranging from oligoclase to andesine. It exhibits albite and Carlsbad twinning (Fig. 13e), is partially saussuritized, and is commonly fractured. K-feldspar occurs in lesser amounts (A 14.4–15 vol%) and has anhedral to subhedral grains of orthoclase, microcline, and microcline perthite (Fig. 13e), respectively. Quartz (Q 26.7–45 vol%) occurs as anhedral grains interstitial to feldspars, displaying wavy extinction (Fig. 13e). Some grains are granulated and fractured (Fig. 13e). Muscovite is the main mafic mineral that occurs as subhedral flakes, pale to colorless, partially chloritized along cleavage planes (Fig. 13e). A lesser amount of Biotite and Hornblende occurs as subhedral flakes, pale to colorless, partially chloritized. Accessories, zircon, apatite, and opaques are present as scattered inclusions, often enclosed in biotite and feldspar (Fig. 13e). The presence of (myrmekitic, porphyritic, poikilitic, mortar, strained quartz, and granulation textures) (Fig. 13e) in the granodiorite indicates that the rock has undergone significant late-stage cataclastic deformation.

Monzogranite mainly consists of K-feldspar, plagioclase, quartz, and the mafic minerals. K-feldspar (20-32.5 vol%) is identified as orthoclase, microcline, and microcline-perthite. Microcline-perthite appears as subhedral crystals with a larger grain size (4 mm in length), distinguished by cross-hatched twinning intermingled with a net-like perthitic texture (Fig. 13d-f). Euhedral prismatic orthoclase crystals have a medium grain size and display distinctive Carlsbad twinning and a perthitic texture (Fig. 12d-f). Plagioclase is present as subhedral to anhedral prismatic crystals of oligoclase that exhibit albite-pericline (Fig. 12d-f) and albite-carlsbad twinning (Fig. 12d-f). Certain crystals exhibit myrmecitic textures, with quartz excluded as vermicules (Fig. 13d). Quartz appears as subhedral to anhedral crystals with wavy extinction. Certain crystals are stretched and elongated, exhibiting moderate undulose extinction (Fig. 13d-f).

Mafic minerals consist primarily of biotite, hornblende, and muscovite. Biotite appears as subhedral to anhedral flakes that pleochroically range from light brown to deep brown. Certain flakes exhibit foliation along the fractures and are linked to the elongated quartz crystals (Fig. 13d-f), while others show partial alteration with varying degrees and have transformed into chlorite with iron oxyhydroxides removed along the cleavage planes (Fig. 13d-f). Hornblende is the second most abundant mafic mineral, with distinct two-directional cleavage evident in basal-sectioned crystals. Muscovite occurs in lesser amounts, showing low relief and high interference colour (Fig. 13d).

Accessory minerals include apatite, epidote, titanite, allanite, opaque minerals, and zircon. Zircon occurs as colorless prismatic crystals with high relief, commonly displaying pleochroic halos within biotite, suggesting higher U-Th concentrations, and in some cases showing zonation within plagioclase and biotite (Fig. 13d, f). Allanite forms elongated euhedral brown crystals with masked interference colors (Fig. 13f).

**Fig. 14:**
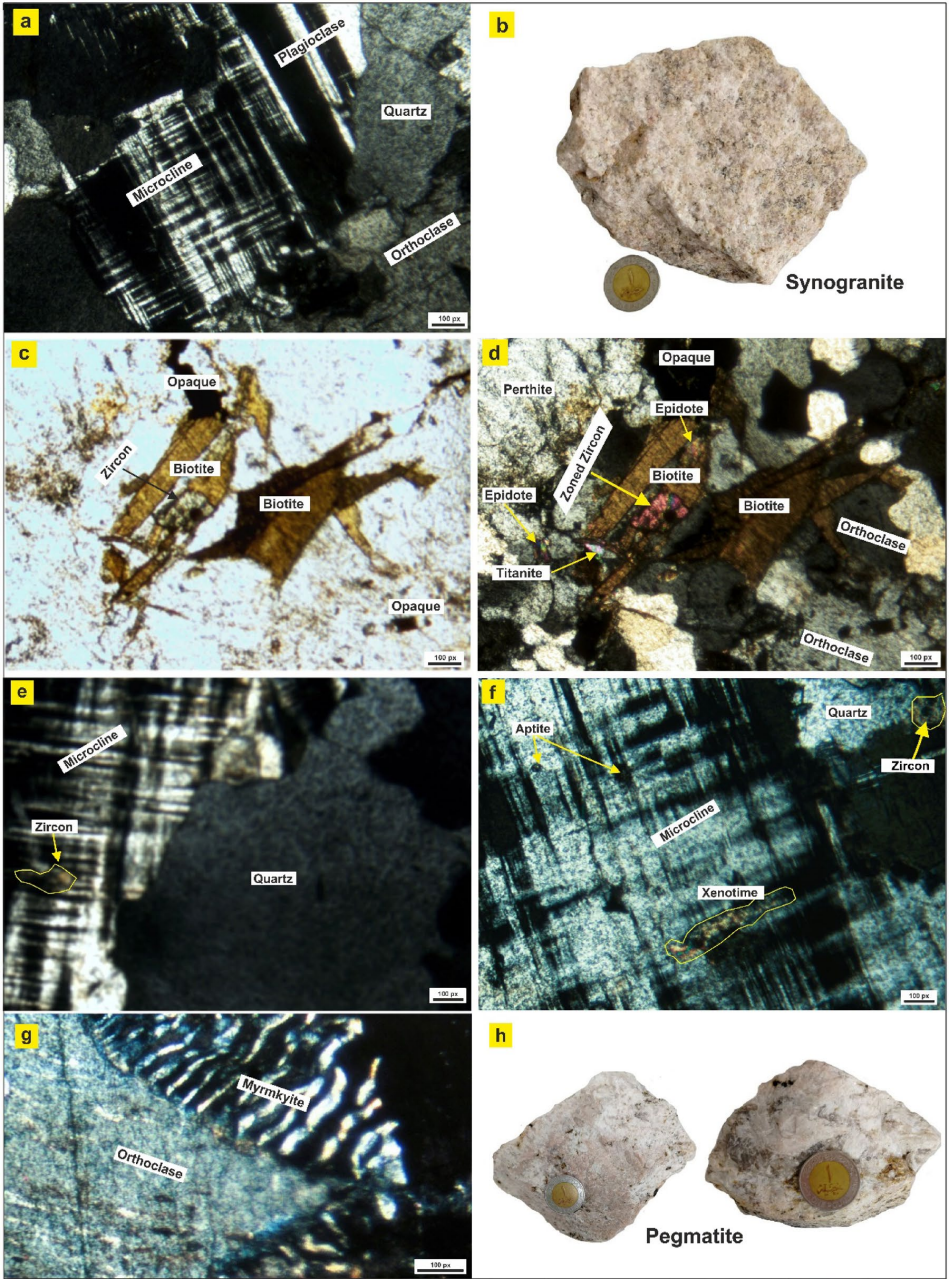
**a**) XPL photomicrograph showing microcline showing crosshatched twinning in association with plagioclase, quartz, and orthoclase, **b**) Hand specimen of syenogranite showing a light-colored, coarse-grained, equigranular texture, **c**) PLL photomicrograph showing biotite flakes containing zircon inclusions and associated opaque minerals, **d**) XPL photomicrograph showing orthoclase, perthite, biotite, epidote, titanite, opaque phases, and zoned zircon, **e**) XPL photomicrograph showing microcline and quartz with zircon inclusions, **f**) XPL photomicrograph showing microcline associated with quartz and accessory apatite, zircon, and xenotime, **g**) XPL photomicrograph showing myrmekitic intergrowths at the orthoclase-plagioclase boundary, **h**) Very coarse-grained, leucocratic mineral assemblages characterize pegmatite hand specimens.

Syenogranite is dominated by quartz, K-feldspar, subordinate plagioclase, and minor biotite. K-feldspar (34.5–42.6%) occurs as euhedral to subhedral megacrysts, commonly forming orthoclase, microcline, and microcline perthite (Fig. 14a). Perthitic and antiperthitic textures are locally developed. Many crystals are slightly strained and fractured. Plagioclase constitutes a minor phase (6.6–14.5 vol%), generally occurring as anhedral to subhedral crystals included within K-feldspar megacrysts (Fig. 14a). It displays albite- and pericline-twinning, is partly saussuritized, and sometimes forms myrmekitic intergrowths with quartz (Fig. 14a). Quartz (50.6–51 vol%) occurs as anhedral to subhedral interstitial grains, displaying undulose extinction. Some grains are fractured (Fig. 14a-d), suggesting post-crystallization deformation.

Biotite is the main mafic mineral and appears as subhedral, pale brown to greenish-brown flakes, infrequently altered to chlorite and iron oxides (Fig. 14c,d). It is frequently associated with feldspar fractures and sometimes encloses zircon inclusions (Fig. 14e). Muscovite occurs in lesser amounts, showing low relief and high interference colour. Collectively, these features indicate that the syenogranites experienced minor cataclastic overprinting, most likely related to post-emplacement tectonic activity.

Accessory minerals such as zircon, epidote, titanite, and zoned zircon appear as large crystals (Fig. 14d), and a large amount of opaques occur as inclusions within feldspar and quartz (Fig. 14d).

Granitic pegmatites are coarse-grained, with individual crystal sizes commonly exceeding 2 cm. They are composed predominantly of K-feldspar, quartz, plagioclase, and minor mafic minerals. K-feldspar occurs as euhedral to subhedral megacrysts, predominantly microcline (Fig. 14e,f), and is frequently associated with microcline perthite. Plagioclase is a lesser constituent, typically occurring as fine-grained inclusions within the megacrysts of potash feldspar (Fig. 14e,f). It displays pericline and albite twinning and is commonly altered to sericite. Locally, plagioclase in association with quartz exhibits myrmekitic intergrowths, indicative of late-stage exsolution processes (Fig. 14g). Quartz occurs as anhedral to subhedral skeletal crystals that are partially enclosed within the megacrysts of microcline and typically display pronounced wavy extinction (Fig. 14e). Biotite and Muscovite are present as irregular flakes intimately associated with plagioclase, commonly filling fractures within the potash feldspar, or as euhedral flakes aligned along fractures in quartz.

Accessory minerals include Zircon, which is also present, typically as fractured crystals coated with iron oxides and often associated with plagioclase (Fig. 14e,f). Xenotime appears as a dashed grey with high relief crystals in polarized plane light and has yellow-violet interference colour (Fig. 14g). Apatite appears as oval inclusions within microcline and biotite (Fig. 14f). Garnets, which are the most characteristic accessory phase. It occurs as usually euhedral to subhedral, equant crystals, colorless to pale yellow. Garnet containing inclusions may form spiral trails indicating syn-kinematic growth.

## Discussion

### Performance and geological implications of automatic lithological mapping using stacked

**Sentinel-2 + ALOS PALSAR Data**.

The classification results demonstrate that Sentinel-2 data, processed through MNF transformation and integrated with multisource datasets, achieve high automated lithological discrimination of the rock units in basement terrains of ANS, G. El-Faraid area, as illustrated in Figs. 6 and 7 and summarized in Tables 3 and 4. SVM generally outperforms RF, particularly in distinguishing spectrally similar granitoid subclasses, reflecting its ability to handle complex, nonlinear class boundaries in high-dimensional feature space.

Classification performance varies significantly among lithological units (Tables 3 and 4), reflecting differences in spectral distinctiveness and mineralogical composition. Wadi deposits (WD) and tonalite–granodiorite (TG) exhibit strong separability due to their distinctive spectral signatures, whereas medium-grained biotite monzogranite (MGr) shows consistently lower performance. This reduced separability is attributed to overlapping quartz–feldspar–biotite assemblages shared with adjacent granitoid units, particularly TG and syenogranite (SG), as reflected in the class-wise performance patterns shown in Tables 1 and 2. These spectral similarities result in frequent misclassification of MGr, as also evident from the confusion patterns illustrated in Figs. 6 and 7, and are further intensified by hydrothermal alteration and surface weathering effects that reduce spectral coherence.

Integration of ALOS SAR data with MNF-transformed optical data produced significant improvements, particularly for Lithologically ambiguous units such as MGr (Tables 3 and 4). This improvement reflects the contribution of L-band SAR backscatter in capturing surface roughness, structural fabric, and textural variability that are not represented in optical reflectance data (Figs. 6 and 7). In contrast, Lithologically distinct units such as WD and TG show only marginal improvement, indicating that optical data alone is sufficient for their discrimination, with limited additional benefit from SAR integration.

The comparative performance of SVM and RF (Tables 3 and 4) highlights the influence of classifier structure on lithological discrimination. SVM achieves superior performance in mixed and spectrally overlapping classes due to its margin optimization in nonlinear feature space, whereas RF demonstrates greater robustness in classes with higher internal variability, such as syenogranite (SG), where ensemble learning reduces sensitivity to spectral noise.

Overall, the integration of multisensor data enhances lithological mapping accuracy and provides improved discrimination of granitoid subtypes, as clearly demonstrated in Figs. 6 and 7; Tables 1 and 2. These findings are consistent with previous studies reporting improved classification performance through optical–SAR data fusion. The Final map of the integrated Sentinel-2 and ALOS PALSAR data is shown in Fig. 18, providing a more reliable representation of the geological framework of the study area.

The demonstrated capacity to discriminate lithological units at > 92% overall accuracy has direct implications for mineral prospectivity mapping in arid shield terrains, where reliable delineation of granitoid subtypes constrains the spatial distribution of host lithologies for rare-metal and rare-earth mineralization associated with highly fractionated granites^56^.

### Hydrothermal alteration and structural control of mineralization

Mineralization in the G. El-Faraid granitoid suite is dominantly controlled by the interaction between progressive magmatic differentiation, deformation phases (D1-D4), and late-stage hydrothermal activity during the terminal evolution of the PAO. The transition from tonalite-granodiorite to monzogranite, syenogranite, and pegmatite reflects advanced fractional crystallization of a calc-alkaline magma, accompanied by enrichment in incompatible and rare elements and minor crustal interaction^58^.

Petrographically, early tonalites and granodiorites are plagioclase-rich (72–79 vol%) and comprise hornblende and biotite, typical of arc-related granitoids^59^. They show deformation-related microstructures such as undulose extinction, grain-size reduction, and mortar textures, suggesting emplacement under brittle to semi-brittle conditions during late-collisional stages. Monzogranites locally display cataclastic fabrics, whereas syenogranites are characterized mainly by hypidiomorphic granular textures with minor deformation overprint, reflecting emplacement during tectonic relaxation. Euhedral zircon crystals with oscillatory zoning indicate crystallization from an oxidized, water-rich melt, consistent with evolved magmatic conditions^60^,^61^.

Structurally, D1 causes thrusts, S1 foliation, L1 lineation, and SE-verging folds in ophiolitic mélanges and arc metavolcanics (Figs. 11a-b). NNW-SSE shortening during intra-oceanic arc collision, manifested in D2, coincided with the emplacement of tonalite and granodiorite during late collisional stages. The sinistral NW-SE shear zones in D3 are considered the major channels for the ascent and emplacement of differentiated granitoids and pegmatites under transtensional to transpressional conditions (Figs. 11e and f). The late D4 brittle deformation resulted in E-W dextral faults and fracture systems (Fig. 11g,h), which played an important role in enhancing permeability and focused hydrothermal circulation.

Hydrothermal alteration is vigorous in monzogranites, syenogranites, and pegmatites. Petrographic evidence for hydrothermal alteration includes chloritization of mafic minerals, sericitization and kaolinization of feldspars, and hematitization along microfractures and margins of hydrothermal veins. Accessory and secondary minerals such as zircon, monazite, xenotime, allanite, and garnet are indicative of the mobilization and enrichment of U-Th-REE and other incompatible elements during late-to-post-magmatic fluid-rock interactions^62^.

The integration of field evidence, petrographic characterization, and remote sensing interpretation confirms that reactivated NW-SE and NE-SW structural trends (D3-D4) played an important role in magma emplacement, hydrothermal fluid circulation, and pegmatitic and rare-metal mineralization in the G. El-Faraid area.

### Potentiality map utilizing fuzzy-logic overlay

**Fig. 15:**
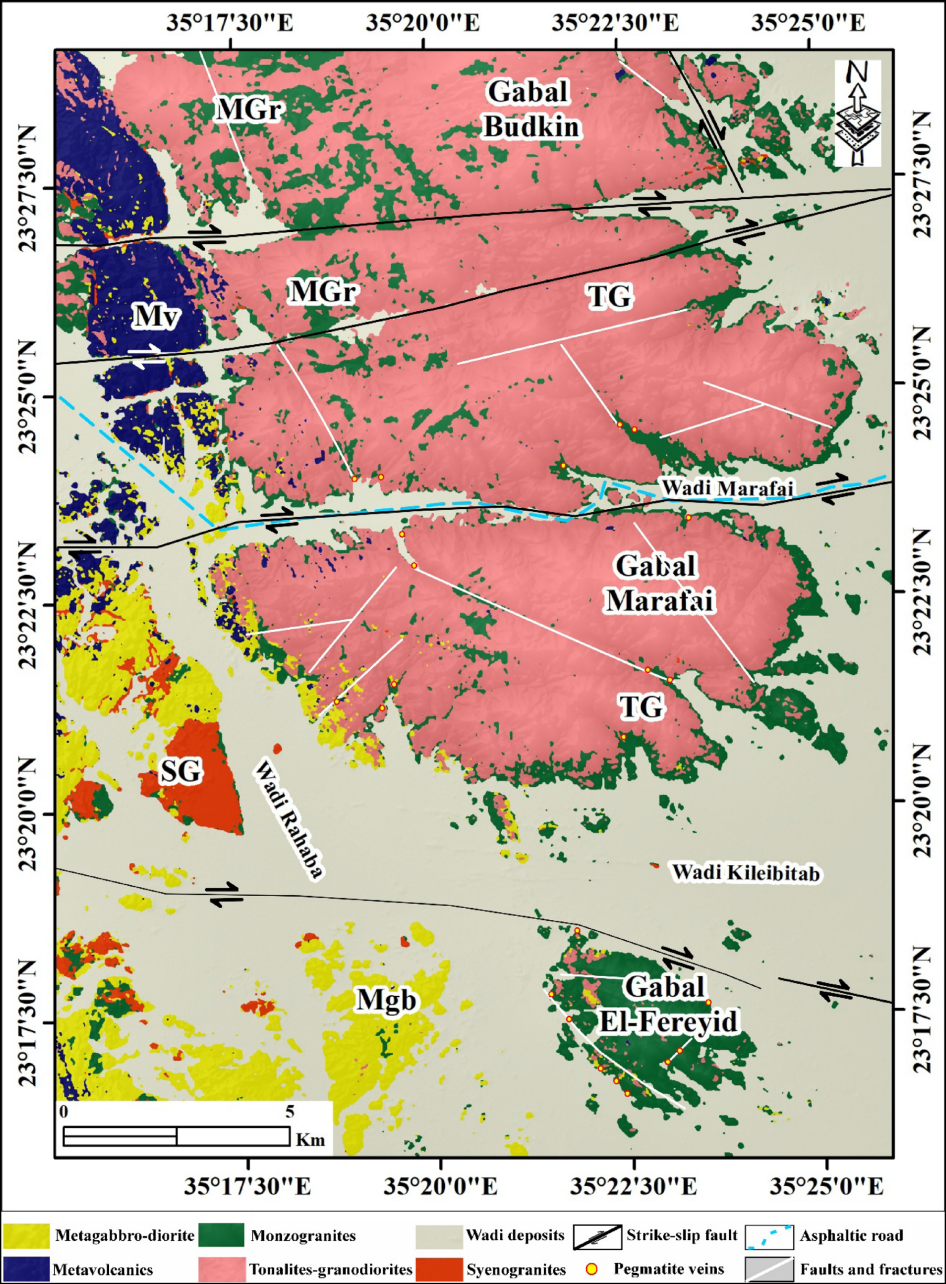
Fuzzy-logic integration of multisource geospatial datasets delineating mineral prospectivity zones within the.

**Fig. 16:**
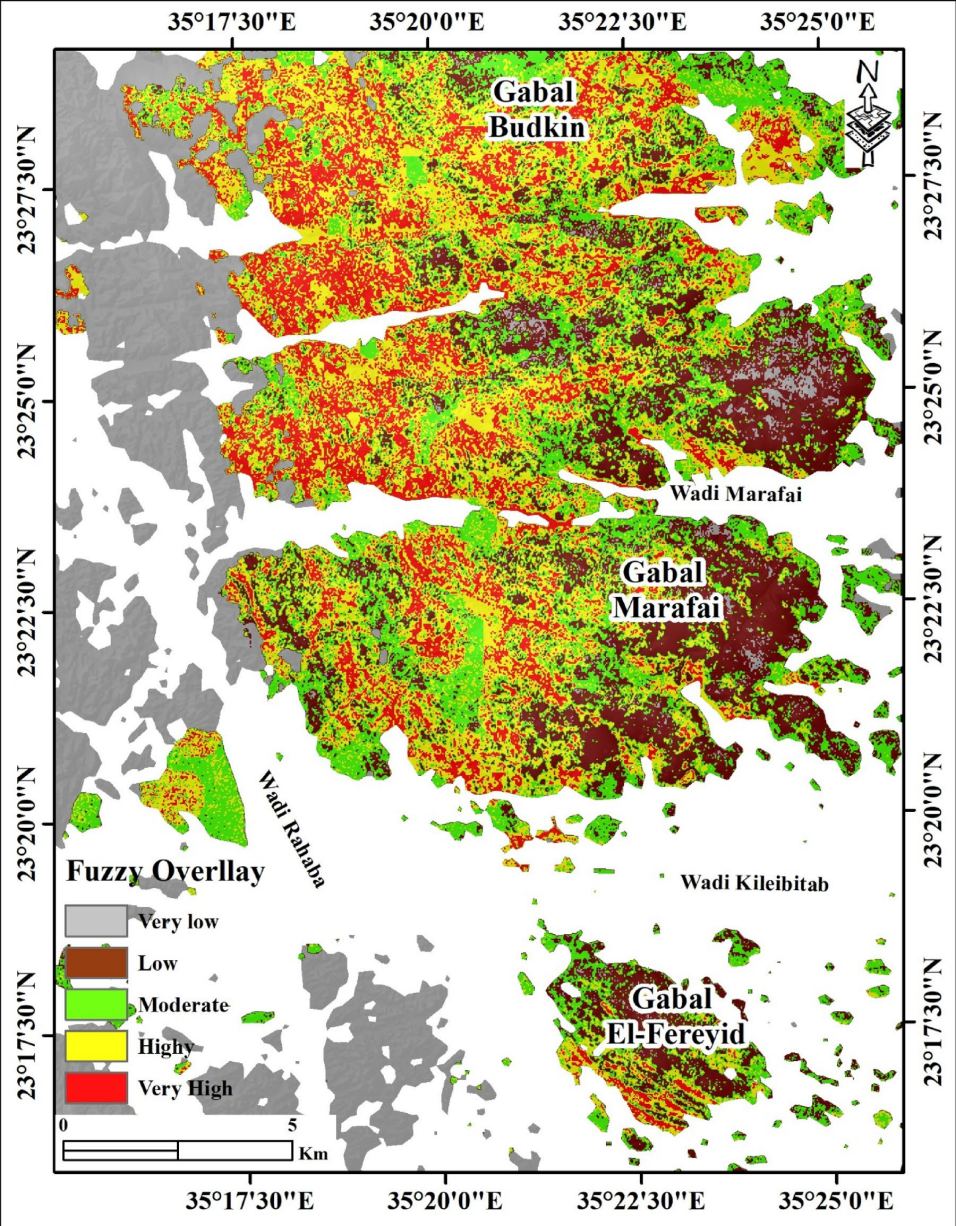
Final geological map of the study area using stacked Sentinel-2 and ALOS PALSAR data. Created by QGIS Desktop 3.36.3 software; https://qgis.org/project/visual-changelogs/visualchangelog336/ and ArcGIS Desktop 10.8. https://www.esri.com/en-us/arcgis/products/arcgis-desktop/overview.

The integration of multisource geospatial datasets through a fuzzy-logic overlay approach constitutes a rigorous and reproducible framework for delineating mineral prospectivity zones within the G. El- Faraid, South Eastern Desert of Egypt (Fig. 15). In this study, multiple evidential layers were compiled to capture the key geological controls on mineralization. These include hydrothermal alteration indices derived from hyperspectral PRISMA data (Fig. 8), structural lineament density maps (Fig. 9b), and automated lithological mapping (Fig. 16). Each thematic layer was standardized through appropriate fuzzy membership functions such as the small membership function for distance-based layers and the logistic membership function for continuous geochemical variables transforming heterogeneous datasets into a common continuous scale ranging from 0 (non-favorable) to 1 (highly favorable). This normalization procedure eliminates bias arising from differing data units and dynamic ranges, while preserving the relative contribution of each predictor variable to the overall prospectivity assessment^63^–^67^.

The fuzzy gamma operator ($\:\gamma\:$) was subsequently employed to combine the standardized thematic layers. This operator is expressed as:

$$\:\mu \:_{{{\mathrm{combination}}}} = \left( {\prod {\:_{{i = 1}}^{n} } \mu \:_{i} } \right)^{{1 - \gamma \:}} \cdot \:\left( {1 - \prod {\:_{{i = 1}}^{n} } (1 - \mu \:_{i} )} \right)^{{\gamma \:}}$$

where $\:{\mu\:}_{i}$ denotes the fuzzy membership value of the *i*th evidence layer and $\:\gamma\:$ is a parameter between 0 and 1 that balances the compensatory effects of the fuzzy algebraic product and fuzzy algebraic sum. This approach enhances zones where multiple favorable criteria spatially coincide particularly areas characterized by both intense hydrothermal alteration and high fracture density while suppressing isolated anomalies that are more likely related to background lithological variability or noise. The gamma value was optimized to maximize the spatial correlation between high fuzzy index values and known mineralization indicators, following established best practices for knowledge-driven mineral prospectivity mapping.

The fuzzy gamma operator was subsequently employed to combine the thematic layers, balancing the compensatory effects of fuzzy algebraic sum and product. This approach enhances zones where multiple favorable criteria spatially coincide particularly areas characterized by intense hydrothermal alteration and high fracture density while suppressing isolated anomalies likely related to background lithological variability. The resulting potentiality map (Fig. 15) reveals a heterogeneous distribution of favorability classes categorized into very low, low, moderate, high, and very high potential zones.

G. El-Faraid area, South Eastern Desert, Egypt. Evidential layers standardized through fuzzy membership functions (0–1 scale) were combined using the fuzzy gamma operator. Very high potential zones (red) cluster along structurally controlled NE–SW and NNW–SSE corridors at eastern Gabal Marafai and Gabal El-Fereyid, coinciding with granite contacts and fault intersections. High potential zones (yellow) surround Gabal Budkin alteration halos, while moderate (green), low (brown), and very low (grey) classes reflect background lithological variability and wadi alluvium (Wadi Rahaba, Wadi Marafai, Wadi Kileibitab). Created by ArcGIS Desktop 10.8. https://www.esri.com/en-us/arcgis/products/arcgis-desktop/overview.

Spatially, very high potential zones are predominantly concentrated along the eastern and southeastern sectors of Gabal Marafai and extend toward Gabal El-Fereyid. These areas coincide with structurally controlled corridors trending predominantly NE–SW and NNW–SSE, consistent with the regional tectonic fabric of the Central Eastern Desert^50^. The strong spatial association between high fuzzy index values and mapped fault intersections suggests that brittle deformation zones acted as conduits for hydrothermal fluid migration, promoting alteration and metal precipitation. The clustering of high favorability pixels near granite contacts further supports a magmatic-hydrothermal model, whereby granitoid intrusions functioned as both heat engines and sources of mineralizing fluids.

In contrast, moderate to high potential classes surrounding Gabal Budkin are spatially associated with alteration halos developed along lithological boundaries and fracture swarms. These zones may represent peripheral hydrothermal systems or structurally remobilized mineralization. The diffuse distribution of moderate values across the central parts of the study area likely reflects overlapping but less intense alteration signatures, possibly indicating distal or overprinted hydrothermal processes.

#### Exploration target zones

Based on the classified potentiality map, four principal exploration target zones are delineated:

**Zone A: Eastern Gabal Marafai**.

This zone is characterized by extensive very high fuzzy index values corresponding to dense structural intersections and pronounced hydrothermal alteration signatures. The coincidence of potassic and phyllic alteration indicators with high lineament density suggests a structurally focused hydrothermal system. The spatial coherence of multiple favorable criteria identifies this area as a prime target for detailed structural mapping, geochemical sampling, and subsurface geophysical surveys.

**Zone B: Gabal El-Fereyid**.

The southern cluster of high to very high potential aligns with major shear zones and granitoid margins. Gabal El-Fereyid is known for hosting monzogranite and pegmatite-associated rare-metal mineralization, with radiometric studies recording eU contents up to 149 ppm and eTh contents up to 375 ppm in anomalous pegmatite samples. The spatial pattern implies strong tectono-magmatic control, likely related to late-stage intrusive phases and associated fluid circulation. The persistence of high fuzzy scores along contact zones indicates potential for polymetallic or rare-metal mineralization, particularly where albitization or silicification is observed^8^,^18^.

**Zone C: Central Corridor (Marafai–Budkin)**.

A NE–SW-trending belt of moderate to high potential reflects structurally mediated fluid pathways connecting the main granitoid bodies. Although alteration intensity is comparatively lower than in Zones A and B, the continuity of favorable signatures along this corridor suggests the possibility of blind or structurally offset mineralization at depth. The structural trends in this zone are consistent with ENE and NNW fault sets documented throughout the study area^68^.

**Zone D: Northern Gabal Budkin**.

Scattered high-potential patches correspond to localized alteration zones along fracture swarms within and around the Gabal Budkin granitoid. These may represent satellite hydrothermal centers or remobilized mineralization associated with late brittle deformation. Targeted ground validation and detailed geochemical sampling are required to confirm their economic significance.

### Limitations

Despite the effective integration of remote sensing, field observations, petrographic analysis, and structural investigations, several limitations are worth noting. Without systematic geochemical sampling, the study mainly relies on hyperspectral and radar data along with structural interpretation; as a result, the identified mineralized zones are potential targets rather than verified ore deposits. Furthermore, subsurface mineralization cannot be directly resolved by these techniques, and the spatial and spectral resolution of the remote sensing datasets may restrict the identification of small-scale, narrow hydrothermal alteration zones. Limited surface exposure, weathering effects, and overprinting by multiple deformation events can affect structural interpretations of shear zones, dike swarms, and deformation phases. Environmental effects are also inferred indirectly, as the research lacks a quantitative evaluation of environmental impacts, including the geochemical mobility of potentially harmful elements and environmental risks associated with mineral mining. Despite its hyperspectral capability, PRISMA’s 30 m spatial resolution limits the detection of narrow alteration veins and sub-pixel mineralogical heterogeneities that commonly characterize vein-hosted mineralization systems. Lastly, the chronological relationships between tectonic evolution, magmatic emplacement, and mineralization are still poorly defined owing to insufficient data sets based on accurate and current geochronology. In addition, the predictive potential map does not incorporate stream sediments, soils, and bedrock geochemical datasets, thereby limiting the map’s capability to differentiate between economic mineralization and other types of alterations like pyritization and hematitization.

## Conclusions

1- The Neoproterozoic basement rocks of G. El-Faraid had a polyphase tectono-magmatic evolution that was related to the PAO and post-orogenic stages.

2- The application of machine-learning algorithms (SVM and RF) to enhanced MNF-transformed Sentinel-2 and combined Sentinel-2–ALOS PALSAR datasets offers an accurate and efficient approach for lithological discrimination up to 92.67% and for mapping six hydrothermal alteration zones, including argillic, clay, ferrous silicates, ferrugination, hydroxyl, and phyllic within complex granitic terrains.

3- The structural evolution of the study area reflects a progressive tectonic event: beginning with NNW–SSE shortening during intra-oceanic arc collision (D1), which produced thrusts, S1 foliation, and stretching lineations, followed by continental collision (D2), generating penetrative NNW–SSE folds and enabling tonalite–granodiorite emplacement under active tectonics. Subsequent sinistral strike-slip shearing along the NFS (D3) controlled the emplacement of monzogranites, syenogranites, and pegmatites, while late brittle deformations (D4) produced E–W dextral faults and fracture networks that improved fluid circulation, hydrothermal alteration, and structural reactivation, thereby localizing magma ascent, vein emplacement, and hydrothermal mineralization.

4- Rare-metal mineralization is localized in areas of intense rare-metal mineralization along NW-SE and NE-SW trending shear zones.

5- Petrographic and microstructural data suggest magmatic crystallization, followed by post-emplacement deformation in tonalite-diorite and granodiorite.

6- The integration of field data, petrography, and remote sensing data suggests the conclusion that structural architecture was the dominant control on magmatic and hydrothermal systems.

7- The fuzzy-logic overlay approach effectively integrated multisource geological datasets to delineate structurally controlled hydrothermal mineralization zones, suggesting high-priority exploration targets within the study area.

## Data Availability

All the data derived from this research are presented in the enclosed tables and figures.
